# Latencies of Pulsed Distortion-Product Otoacoustic Emissions and Their Relation to Auditory Brainstem Responses

**DOI:** 10.1007/s10162-025-01019-7

**Published:** 2025-11-26

**Authors:** Ernst Dalhoff, Dennis Zelle, Katharina Bader

**Affiliations:** 1 Department of Otolaryngology, Head and Neck Surgery, Eberhard-Karls-University Tübingen, Elfriede-Aulhorn-Str. 5, Tübingen, 72076 Baden-Württemberg Germany; 2 Earlab GmbH, Karlstr. 3, Tübingen, 72072 Baden-Württemberg Germany

**Keywords:** Group delay, Cochlear tuning, Minimum-phase principle, Traveling wave, DPOAE, Test-retest reliability

## Abstract

**Purpose:**

To assess system properties of the human auditory system, such as cochlear gain, frequency selectivity, and their dependence on frequency and level, it is essential to examine the interrelation of various readouts. By measuring and analyzing otoacoustic emission (OAE) and auditory brainstem response (ABR) latencies, among others, predictions of cochlear models and applicability of properties such as the minimum-phase principle, level dependence of latencies, or related changes of the gain of a presumed positive-feedback mechanism can be investigated.

**Methods:**

Here, we present measurements of the latency of the nonlinear-distortion component of pulsed distortion-product otoacoustic emissions (DPOAE) ($\boldsymbol{f_2}$ = 1–14 kHz, $\boldsymbol{L_2}$ = 25–85 dB SPL) in 20 ears (12 female, 8 male). This yields a direct estimate of intracochlear traveling-wave build-up by recording the time elapsed between the $\boldsymbol{f_2}$ primary stimulus and the distortion-product pulse response. Thus, this technique does not require deriving latency from phase gradients of the coherent-reflection component of different frequencies, as is done using swept-tone DPOAE or SFOAE.

**Results:**

At low stimulus levels ($\boldsymbol{L_2}$ = 35 dB), DPOAE latency was 13 ms at $\boldsymbol{f_2}$ = 1 kHz, exponentially to  2 ms at $\boldsymbol{f_2}$ = 12–14 kHz. In periods of the corresponding frequency, this rose from $\approx$13 periods at 1 kHz to $\ge$25 periods above 6 kHz. Between 3 and 6 kHz, latency showed a steeper rise, departing from a pure exponential relation. Level dependence of latencies varied among subjects, with changes ranging from –2 to –12% per 10 dB level increase. Test-retest reliability of latency determination with pulsed DPOAE was excellent.

**Conclusion:**

For frequencies above 1 kHz and up to 14 kHz, OAE latency data align with a scaling law of $\approx$0.3 dB/dB. A transition region between 3 and 6 kHz shows scaling in some ears approaching 1 dB/dB, violating local scaling symmetry. Although comparison with ABR literature reveals some unresolved discrepancies, latencies of pulsed DPOAE allow a way to estimate cochlear tuning properties.

**Supplementary Information:**

The online version contains supplementary material available at 10.1007/s10162-025-01019-7.

## Introduction

Direct measurements, such as auditory-nerve single-fiber recordings or intracochlear vibration measurements to acquire information about tuning bandwidths of cochlear filters, are not feasible in humans. Therefore, derivation of latencies of cochlear traveling waves using non-invasively accessible metrics are key to transfer evidence about the signal processing of hearing gained from animal experiments to human auditory function (and dysfunction) [1]; also refer to Fig. 1 of [2]. The passive mechanical function of the cochlea may be described as a locally tuned spectrum analyzer capable of transducing sounds of about 60 to 120 dB SPL within a broad species-dependent frequency band into the dynamical range within which the inner hair cells can produce action potentials. According to at least one, if not the prevailing, view, by adding the nonlinear cochlear amplifier [3], more signal gain and thus lower thresholds are achieved, and the added gain is accompanied by higher frequency resolution and longer signal latencies [4, 5].

**Fig. 1:**
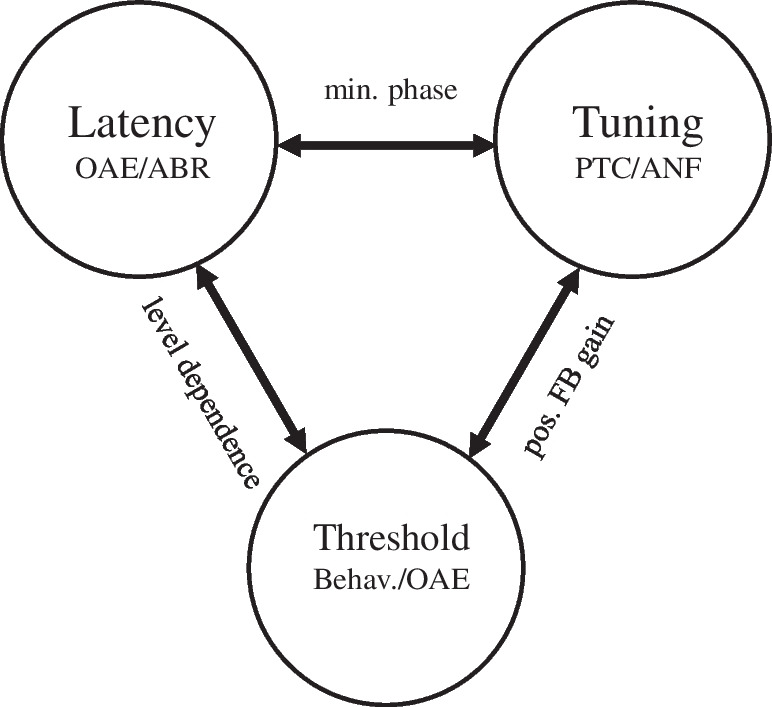
To assess system properties of the human auditory system, such as cochlear gain, frequency selectivity, and their dependence on frequency and level, it is essential to examine the interrelation of various readouts. By measuring and analyzing otoacoustic emission (OAE) and auditory brainstem response (ABR) latencies, auditory thresholds, and psychoacoustic frequency tuning curves (PTC), predictions of cochlear models and applicability of properties such as the minimum-phase principle, level dependence of latencies, or related changes of the gain of a presumed positive-feedback mechanism (pos. FB gain) can be investigated

Within a framework of linear minimum-phase systems, the latency of a signal to reach its characteristic place in the cochlea bears a fixed relationship to frequency tuning [6–9]: sharper tuning is achievable only with longer latencies. Animal experiments have demonstrated that basilar-membrane and auditory-nerve tuning curves show almost identical tuning curves [10–12]. This finding directly connects cochlear latency to bandwidth (or Q-factor) of psychophysical tuning curves (PTC; see Fig. 1), which can be measured in humans [13–17].

In addition, the current understanding of the cochlear amplifier and related experimental work in mammals has established the principle that the more gain the cochlear amplifier adds pre-neurally, the sharper the frequency tuning becomes, thus relating frequency tuning to hearing threshold [8]. This latter relation is also reflected in mathematical models of cochlear traveling-wave generation that include an active feedback mechanism [4, 18–20]. For instance, in a cochlear model that includes a realistic middle-ear representation and is based on published anatomical and material parameters of the human ear — where cochlear gain and tonotopic mapping have been adjusted to reflect characteristics previously reported for the human cochlea [15, 21] — there is good agreement in frequency tuning, measured as $Q_{\textrm{10dB}}$, with psychoacoustical values determined by Vinay and Moore [22] from 0.5 to 4 kHz, and also [17] up to 8 kHz ([20], cf. his Fig. 4.21).^1^

Besides its frequency dependence, the latency of human neural responses also depends nonlinearly on stimulus level [23, 24], their Fig. 3. The level dependence of latencies is thought to be predominantly of cochlear origin [25, 26]. Comparisons between tone-burst evoked auditory brainstem response (ABR) wave V and otoacoustic emission delay indicate that the level dependence behaves qualitatively similar in both measures [26–28]. With respect to the frequency dependence, the purely cochlear origin of nonlinearity is supported by the fact that compensating for cochlear delay by applying optimized chirp stimuli has been demonstrated to maximize ABR responses [29, 30].

Given that direct intracochlear vibration measurements and auditory-nerve single-fiber recordings in humans are not feasible, indirect measurements are essential for understanding the function of the cochlear amplifier and validating models that relate tuning, gain, and bandwidth of human cochlear filters and their variation with frequency and level. This argument is illustrated in Fig. 1.

Studies of frequency-dependence of OAE latencies in both animals and humans have focused on deviations from a simple power law that can be described by a single exponent only [31, 32]. Specifically in humans, a “break” in continuity has been suggested in latency measurements of OAE at around 1 kHz [32], and a second break at around 2.5 kHz [33]. Several investigations, including the latter two, base their latency estimates on the group delay of stimulus-frequency otoacoustic emissions (SFOAE), derived from the phase gradient.

Measuring the phase gradient requires measurements at different frequencies, typically achieved by sweeping the stimulus. Deriving latency from a steady-state representation of a signal, rather than from transient signals, can introduce specific challenges, particularly in individual measurements (see Appendix A).

Apart from that, studies including ABR measurements [26–28] do not appear to clearly support all reported breaks in continuity.

Recently, in a test-retest study, we gathered pulsed DPOAE data in ten normal-hearing subjects in the frequency range 1–14 kHz. From these data, the latency of the nonlinear-distortion component of the DPOAE can be directly obtained in the time domain. Here, we present the frequency and level dependence of pulsed DPOAE latency and compare the results with previously published ABR and OAE data.

**Fig. 2:**
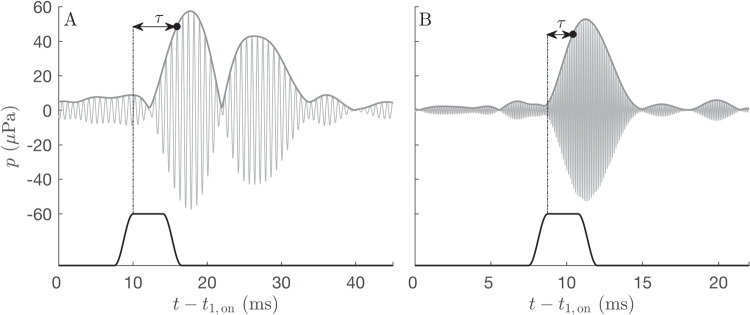
Time courses of short-pulse DPOAE signals relative to the onset of the $f_1$-pulse for $f_2$= 3 kHz (**A**) and $f_2$ = 13 kHz (**B**). The latency of the nonlinear-distortion component $\tau$ is defined as the time between the point when the $f_2$ pulse (bottom) assumes its full amplitude after turning on the $f_2$ stimulus tone and the extraction of the amplitude of the nonlinear-distortion component. $f_2$ pulses were shaped as a Tukey windows with frequency-specific full widths at half maximum according to $T_{\textrm{2,HW}}(f_2) = 13.07/f_2$, and the value $T_{\textrm{2,HW}} (f_2)$ = 3.27 ms at $f_2$ = 4 kHz defined the lower boundary for the full widths at half maximum for higher frequencies to enable narrow band-pass filtering [38]. The total recording time for all short-pulse DPOAEs at 14 frequencies with 10 levels was 6 min

**Fig. 3:**
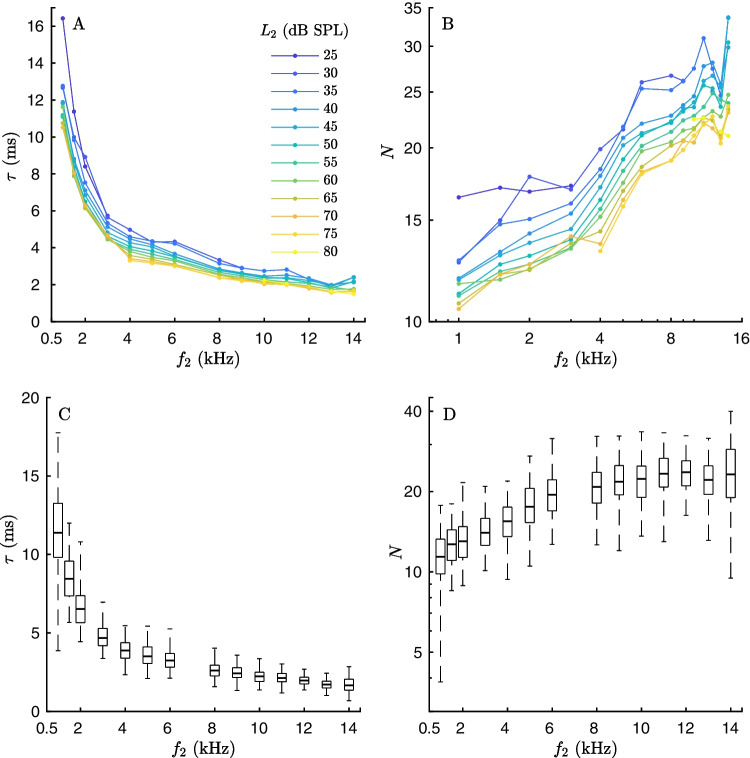
Characteristics of pulsed DPOAE latencies. **A** Mean values of latencies, expressed in milliseconds as a function of the linearly scaled frequency, for the respective stimulus levels $L_2$ (colored lines) of all sessions. The data indicate that latency decreases exponentially with increasing frequency. **B** Mean values of latencies, expressed in periods *N* of the corresponding $f_2$, for the respective levels $L_2$ (colored lines) of all sessions, and plotted on a double-logarithmic scale. This representation approximates tonotopic scaling. An increased rise in the slope between 3 and 6 kHz is observed. Additionally, there appears to be a break at 1.5 kHz, below which the slope is also steeper. **C** Median DPOAE latency collated across all $L_2$ and sessions, expressed in milliseconds. **D** Median latency collated across all $L_2$ and sessions, expressed in periods. One boxplot per frequency. Boxes: IQR from the first to the third quartile. Error bars: 95%-range

## Material and Methods

### Subjects and Study Design

DPOAE data presented here originated from a published test-retest study that focused on properties other than latency. Ten normal-hearing subjects (four-frequency pure-tone average 0.5–4 kHz <20 dB HL, aged 21–56 years) participated in seven test sessions within three months. Only two ears presented a threshold of more than 20 dB HL at 8 kHz, and 2/3 of the ears maintained thresholds below 20 dB HL up to 16 kHz. The thresholds are shown in Figure 1 of [34]. The next section briefly explains the DPOAE paradigm used in this study. The study was approved by the Ethics Committee of the University of Tübingen (265/2018BO1) in accordance with the Declaration of Helsinki for human experiments. Informed consent and a data privacy statement were obtained from each subject for experimentation of human subjects. For further details, the reader is referred to [35].

### DPOAE Acquisition

DPOAEs were measured using two Etymotic ER-10C probes (Etymotic Research, Elk Grove Village, IL, USA), two National Instruments data acquisition cards (NI PCI 6733, NI PCI 4472, National Instruments, Austin, TX, USA), and custom-built software (LabVIEW Version 17.0, National Instruments, Austin, TX, USA), in both ears. Calibration was performed in-ear, and sound-pressure level at the tympanic membrane was estimated by using a correction based on an artificial ear compliant with IEC 60318-4. Automated DPOAE signal analysis was performed using custom-made software created with MATLAB (Version 9.6, The MathWorks, Natick, MA, USA).

Pulsed DPOAEs were recorded for $1 \le f_2 \le 14$ kHz with $f_2/f_1=1.2$. Time responses of the isolated $2f_1 - f_2$ distortion product were extracted using the primary-tone phase variation technique [36] by shifting the $f_2$ and $f_1$ stimulus tones in consecutive blocks by $90^\circ$ and $180^\circ$, respectively, with additional digital filtering to improve the suppression of the stimulus tones. These DPOAE pulse responses comprise, if present, both the nonlinear-distortion and the coherent-reflection component of the DPOAE. Seven stimulus pulse pairs were organized in an interlaced arrangement within one block, and two such blocks are used to cover 14 frequencies. We used an $f_2$ short-pulse stimulus, i.e., the $f_1$ pulse was presented for a duration between 20 and 40 ms, whereas the duration of the $f_2$ pulse was chosen to be shorter dependent on frequency, to separate the nonlinear-distortion and the coherent-reflection pulse responses in the time domain.

This timing ensures that the nonlinear-distortion component already decays before the longer-latency coherent-reflection component rises considerably. The low-latency, nonlinear-distortion component of the DPOAE was extracted using an onset-decomposition (OD) algorithm, which sampled the pulse-response waveform close to the point of having reached the steady state of the nonlinear-distortion component, $t_{\textrm{OD}}$, thus avoiding interference due to the coherent-reflection component [37].

Ten different stimulus levels were presented in 5-dB steps, with the lowest $L_2$ level ranging from 25 to 35 dB SPL depending on frequency. The stimulus levels of the first primary, $L_1$, were chosen, where possible, as the individually optimal combinations with $L_2$, based on the projection of the ridge of a separately measured DPOAE level map to the $L_2, L_1$ plane [38]. This can be understood as stimulating along an individually optimum path, as compared to using a group-optimized path such as the scissors paradigm [39]. Pulse responses had to meet a signal-to-noise ratio criterion (SNR $\ge 10$ dB) and pass a $\chi ^2$ test qualifying its variance in comparison to the superimposed noise.

### Determination and Modeling of Latencies

Latencies are determined from the recorded waveforms in the time domain as $\tau = t_{\textrm{OD}} - t_{2,\textrm{ss}}$, where $t_{2,\textrm{ss}}$ is the time when the $f_2$ pulse reaches its steady state (i.e., at the end of the rising ramp; see Fig. 2). For a detailed description of the OD technique, refer to [37], their methods D.3. Thus, the latencies presented here derive directly from the isolated nonlinear-distortion component. The cosine-shaped ramps as well as the widths of the$f_2$ pulses depended on frequency up to 4 kHz, and were constant above, as shown in Fig. 9. The “Possible Influence of Ramp Durations and Pulse-Width Choice” section discusses potential consequences of this choice.

Latency is presented in two ways: First, latency is plotted in linear units, showing time vs. frequency (Fig. 3A). Second, latency is converted from time to periods of the second primary frequency $N(f_2)=\tau (f_2)\,f_2$, plotted on a double-logarithmic scale (Fig. 3B). This conversion transforms the frequency-dependence of any power-law relation between latency and frequency into a straight line, which is advantageous, because it has been established that cochlear latency as well as ABR latency follows at least roughly a power-law relation, and thus, the fitting problem can be reduced to linear regression. Moreover, any deviation from a straight line with zero slope directly indicates the departure from ideal scaling symmetry of the cochlea. The presentation of periods in dB is defined as $\Gamma$:

1 $$\begin{aligned} \Gamma (f_2) = 20\log _{10}(N(f_2)) \end{aligned}$$

**Fig. 4:**
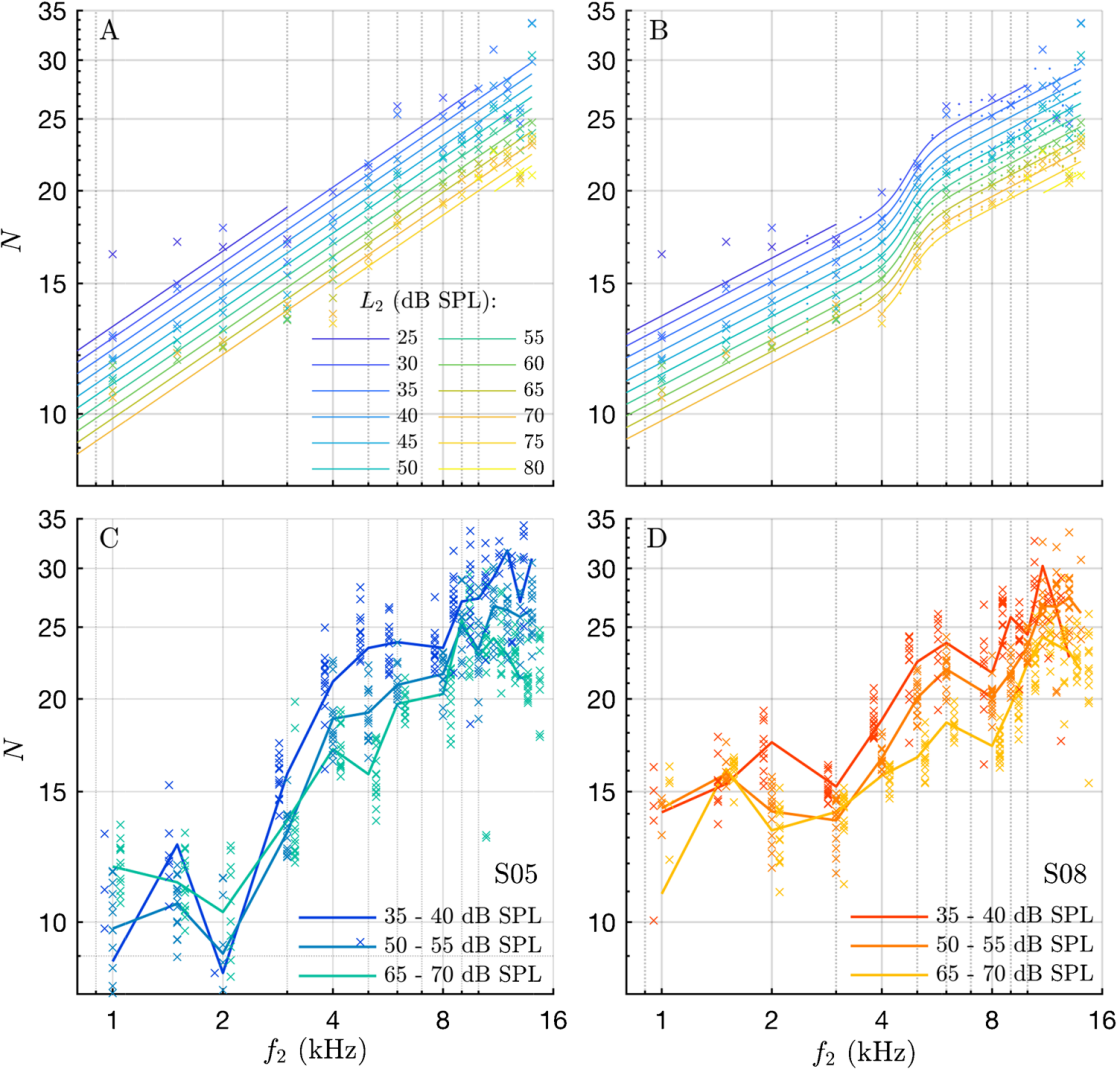
Upper row: Model Fits of log-log-scaled latency period functions to the mean values of all ears and sessions. **A** Fit of a single exponent model [Eq. 2], using only the first three terms with parameters $c_1$, $c_2$ and $c_3$). **B** Fit of a single exponent model, but modulated by a tanh-function to account for the steeper latency rise between 3 and 6 kHz [Eq. 2]. To obtain a function with a smooth transition, the frequency resolution of the experimental points was increased by linear interpolation (dots represent interpolated data points; see Methods). Each color represents a different stimulus level, ranging from 25 $\le L_2 \le$ 80 dB SPL (from dark blue to yellow). The starting and ending points of the model fits at intermediate frequencies are due to some levels being used only within certain frequency ranges (see Methods). Crosses mark the mean values across ears and sessions, depending on frequency and level. Bottom row: Examples of inter-subject variability. **C**, **D** Two examples of ears S05 and S08 illustrating the inter-subject variability in the shape of the frequency dependence. Each curve shows the mean value of the periods for two stimulus levels 5 dB apart for low, medium, and high level stimulation over all sessions (see legend). The “breaks” in frequency dependence differ clearly: in S05 (**C**), there is a transition region extending 2–4 kHz and leading to approximately a doubling of periods (thus reaching approximately 1 dB/dB), whereas in S08 (**D**), the transition region extends 3–6 kHz and results in approximately a 1.5 time increase in periods. For reason of clarity, the crosses for the low and the high level groups are slightly shifted with respect to frequency. These panels highlight the individual differences in frequency dependence of latencies across subjects

Following this choice, the model function ($\tilde{\Gamma }$) is defined as a power-law function plus an additional perturbing function. This augmentation is intended to mimic the characteristic departure observed in a transition region from a pure power law, as suggested by our data:

2 $$\begin{aligned} \tilde{\Gamma }(f_2)= &   c_1 + c_2 20\log _{10}\left( \frac{f_2}{f_\textrm{ref}}\right) + c_3\left( \frac{-L_2}{L_\textrm{ref}}\right) \nonumber \\  &   + \frac{c_4}{2} \left( \tanh \left( c_5 20\log _{10}\left( \frac{f_2}{c_6}\right) \right) \right) \end{aligned}$$

where $$c_i,\; i=1...6$$, are the model parameters, $$f_2$$ the frequency in kHz, $$L_2$$ the level of the second primary in dB SPL, $$f_\textrm{ref}=1$$ kHz, and $$L_\textrm{ref}=100$$ dB SPL. The first three parameters $$c_{1...3}$$ correspond to the parameters $$b,\, d,$$ and *c*, of the formulation of [26] and [27], by the formulae $$c_1 = 20 \log _{10}(b)$$, $$c_2 = 1-d $$, and $$c_3 = 20 \log _{10}(c)$$. The model function is fitted to the experimental data using the MATLAB function *lsqnonlin*. Because the fit to the experimental values led to an unreasonably sharp transition covered by the tanh function at around $$f_2$$=4.5 kHz, the frequency resolution of experimental points to be fitted was artificially increased to 500 Hz using linear interpolation to obtain a smooth transition region (see Fig. 4B).

When estimating cochlear delay from ABR wave V latencies for comparison with our DPOAE data, we account for synaptic and wave I-V delay by subtracting $\tau _{\textrm{syn,V}}$ = 5 ms [27, 28]. For the narrow-band wave, I action potential (NAP) latency derived from electrocochleography, Fig. 6 of [7] was digitized (normal-hearing subjects), which shows the NAP delay minus an assumed synaptic delay of $\tau _{\textrm{syn}}=0.8$ ms. To account for the approximately double travel time comprised in OAE latency, ABR derived roundtrip delays are computed with a factor of 2: $\tau _{\textrm{OAE}}$ = 2 ($\tau _{\textrm{ABR,V}}$ - $\tau _{\textrm{syn,V}}$) (and similarly for the NAP). For justification of this simplified choice, the reader is referred to the “Discussion” section (“The Factor of 2 and Whether OAE Are Backpropagated by Compressional or Slowly Traveling Waves”).

### Reliability of Latencies

To quantify intra-subject reliability or test-retest reliability of $\tau$, *N* and $\Gamma$, average absolute differences between test and retest measurements were determined [40]. The test-retest reliability determines the ability of one method to provide similar results when repeated for the same subject under the same experimental conditions. Defining latency in terms of $\Gamma$ leads to a relative test-retest reliability with little frequency dependence (see “Results” section).

For investigating the variability of the level dependence of latency, an additional measure is introduced:

3 $$\begin{aligned} \Delta \Gamma _{\Delta L_2}(f_2) = 20\log _{10} \frac{N (f_2, L_{2,i})}{N (f_2, L_{2,j})} \end{aligned}$$

that calculates the relative latency between DPOAE responses associated with two different stimulus levels, such as $L_{2,i}$ = 35 dB SPL and $L_{2,j}$ = 65 dB SPL. This somewhat arbitrary measure was chosen as a more intuitive alternative of the fit parameter $c_3$. For example, a level difference of $\Delta L_2=30$ dB for the specified $L_2$ values corresponds to $\Delta \Gamma _{30}=0.3\,c_3$.

## Results

Figure 3 shows the mean latency values as a function of frequency and stimulus level across all ears and test sessions. In Fig. 3A, latencies are presented in linear units, i.e., time vs. frequency. Mean latencies range from 10 to 17 ms at 1 kHz and decrease negative exponentially to approximately 2 ms at 14 kHz. Higher sound-pressure levels systematically result in shorter latencies. Figure 3B displays the same data in periods of the second primary frequency $f_2$, *N*, plotted on a double-logarithmic scale.

In this representation, latency demonstrates an almost monotonic and, in terms of $\Gamma (\textrm{log}_{10}(\textit{f}_2))$, roughly linear growth up to 14 kHz, corresponding to a doubling of the periods from 10 to 17 periods at 1 kHz to 22–35 periods at 14 kHz (Fig.  3B). In particular, within a transition region between approximately 3 and 6 kHz, the slope of the period appears consistently steeper compared to both lower and higher frequencies. With respect to level-dependence, it might be noted that it appears more regular at intermediate levels except for the lowest and the highest ones. Particularly at the lowest measured levels, latencies tend to depart towards exceptionally high latencies at frequencies outside the transition region.

Figure 4 presents the mean latencies scaled in periods (crosses), overlaid with two versions of curve fits according to Eq. 2 (lines in Fig. 4A and 4B) along with two examples of individual frequency and level dependencies of DPOAE latency (Fig. 4C and D). Panel A shows the one-exponent fit using only the first three terms of Eq. 2, yielding an exponent of $c_2=0.338$ (see Table 1). When fitting all latencies in linear units of ms as a function of frequency $f_2$ in kHz (as seen in Fig. 3A), without accounting for level-dependence, the exponent of the frequency dependence was $-0.701\pm 0.003$. When scaled in periods, the exponent is 0.299. Panel B displays the fit using all six terms of Eq. 2, resulting in a smaller exponent of $c_2=0.259$. This reduction is attributed to the tanh term in the equation, which accounts for the steeper rise in the mid-frequency region. The complete list of the fit parameters is given in Table 1, all of which were significant at $p\le 0.05$ (two-sided t-test on whether a parameter is different from zero).

The overall quality of the fits is high, the standard deviation of the residuals was $\sigma _{\textrm{lin}}=1.406$, corresponding to 1.18 periods, for the linear fit (three terms), and $\sigma _{\textrm{log}}=1.069$, corresponding to 1.13 periods, for the nonlinear fit (six terms). Relative to the overall mean value of 21.4 periods, the standard deviation corresponds thus to a 17.6% change for the linear fit, and 13.1% for the nonlinear fit according to Eq. 2. The contribution of the 4th term of Eq. 2 (the tanh-term) at $f_2=c_6$, is given by $\frac{1}{2}c_4\,c_5$, yielding 0.57 dB/dB. Thus, the local slope of $\tilde{\Gamma }$ in the transition region is $0.26 + 0.57 = 0.83$ dB/dB, more than tripled as compared to the slope of $c_2=0.26$ found outside the transition region, and more than doubled as compared to the value of 0.338 for the linear fit with only the first three terms of Eq. 2.

**Table 1:** Fitting parameters $c_i$ according to Eq. 2 along with their standard error (SE), for two fits: when using all six terms, and when using only the first three terms of the equation

i	$c_i$	± SE	$c_{i,\, 1...3}$	SE	*b*, *d*, *c*
1	24.9	0.207	23.9	0.223	15.7
2	0.259	0.014	0.338	0.0091	0.662
3	6.283	0.194	6.196	0.353	2.041
4	1.375	0.214			
5	0.823	0.337			
6	4.718	0.134			

$c_{1-5}$ are dimensionless, $c_6$ in kHz. *b, c, d* are the correspondent parameters as used in [26, 27]

**Fig. 5:**
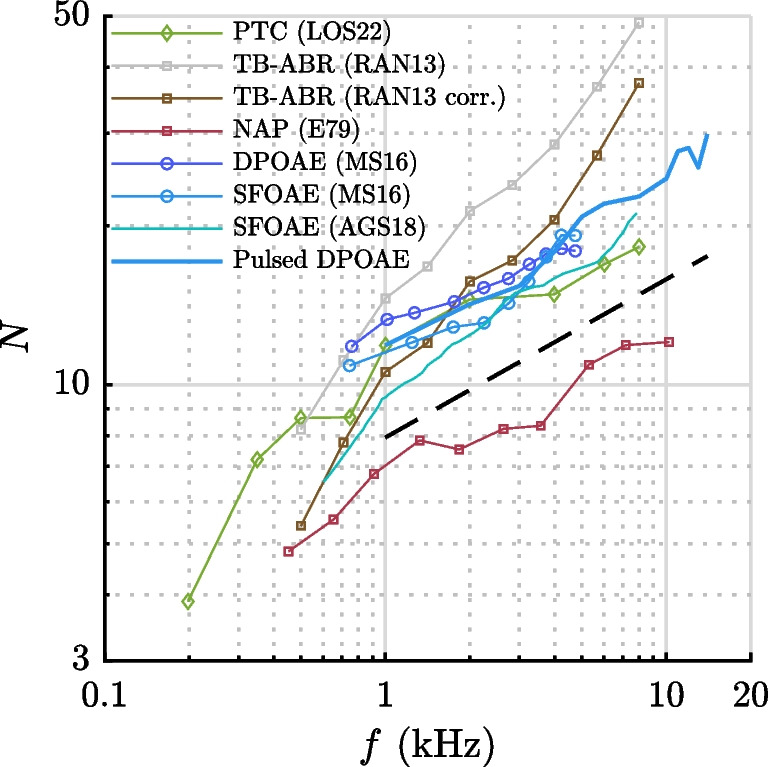
Comparison of the mean values of pulsed DPOAE latencies stimulated at $L_2$=40 dB SPL with exemplary data from existing OAE, group delay, ABR, and PTC literature, scaled as periods of $f_2$ or f in log-log scaling. The general dependence on frequency aligns well with roughly a doubling of periods within the decade of frequency (between 1 and 10 kHz), as indicated by the black dashed line representing a slope of 0.3 dB/dB. The green line represents $Q_{\textrm{erb}}$ data derived from psycho-physical tuning curves (LOS22), the brown and red curves derive from tone-burst ABR wave-V (TB-ABR, RAS13 corr.) and narrow-band action potential data (NAP, E79), respectively. The gray curve (RAN13) shows the uncorrected ABR data (s. text)

The two individual examples of mean latency values as a function of frequency shown in Fig. 4, panel C and D, illustrate the variable occurrence of the transition region, highlighting the inter-subject variability of the frequency dependencies of DPOAE latency. In Fig. 4C and D, latencies from all sessions and two stimulus levels 5 dB apart were averaged to yield a mean value for each ear-frequency combination. Panel C shows the frequency dependence from the left ear of subject S05 with a transition region extending from 2 to 4 kHz, resulting in approximately a doubling of periods. In contrast, panel D displays the frequency dependence from the right ear of subject S08 with a transition region spanning from 3 to 6 kHz.

Figure 5 compares mean latencies of pulsed DPOAE as a function of frequency (blue bold line) with latencies obtained from time-frequency filtered DPOAE (dark-blue circles, MS16 [41]), SFOAE (blue circles, MS16 [41]; cyan circles, AGS18 [42]) for $L_2$ = 40 dB SPL, along with adjusted values from two ABR studies (brown squares, RAN13 corr. Rasetshwane et al. [27], *L* = 40 dB SPL; red squares, E79 [7], *L* = 90 dB peSPL) and one psycho-physical tuning curve study (green diamonds, LOS22 [17], *L*=12 dB SL).

Generally, the frequency dependence observed in all OAE data shown (blue and cyan curves in Fig. 5) for $f\ge$ 1 kHz aligns well with an increase in periods of 0.3 dB/dB (black dashed line). Examining local changes in steepness of the latency growth with frequency reveals some subtle dissimilarities: SFOAE phase gradient data (AGS18 [42]) show a slight reduction in steepness beginning at about 2.7 kHz, and an increase in steepness starting from 5.7 kHz, whereas our data indicate almost contrary behavior. DPOAE data obtained through time-frequency filtering (MS16 [41]) exhibit only a modest steepening of the slope, likely ending around 4.2 kHz, near the upper limit of their measurement range. This characteristic appears to be generally consistent with our findings. Notably, SFOAE latencies based on time-frequency filtering (MS16 [41]) show a steepening beginning at 2.2 kHz and ending at 4.2 kHz, similar to their DPOAE data. In conclusion, the OAE curves from studies investigating latency in the time domain, whether based on DPOAE or SFOAE, are very similar and do not contradict each other regarding changes in local steepness within the frequency range of overlap.

ABR latencies are illustrated by the red and brown curves in Fig. 5. The gray curve shows tone-burst evoked ABR forward delays with frequency-dependent tone-burst rise times (RAN13 [27]) multiplied by two (see the “Determination and Modeling of Latencies” section). From this curve, twice the ramp duration was subtracted to estimate the correspondent group delay (RAN13 corr., brown curve). This curve, derived from 40 dB SPL tone-burst wave-V latencies, exhibits generally higher latencies than the other methods, and a clearly steeper frequency dependence, ending with almost 40 periods at 8 kHz. The red curve represents doubled cochlear latencies derived from narrow-band action potentials (NAP, ABR wave I, E79 [7]), obtained by electrocochleography with 70 $\mu$s clicks and appropriate high-pass masking at stimulus levels of 90 dB peSPL. This method resulted in a frequency dependence that matches the OAE data shown here fairly well. If this curve is adjusted upward by 3 dB to account for the higher SPL, the agreement with respect to absolute values is also reasonable.

**Fig. 6:**
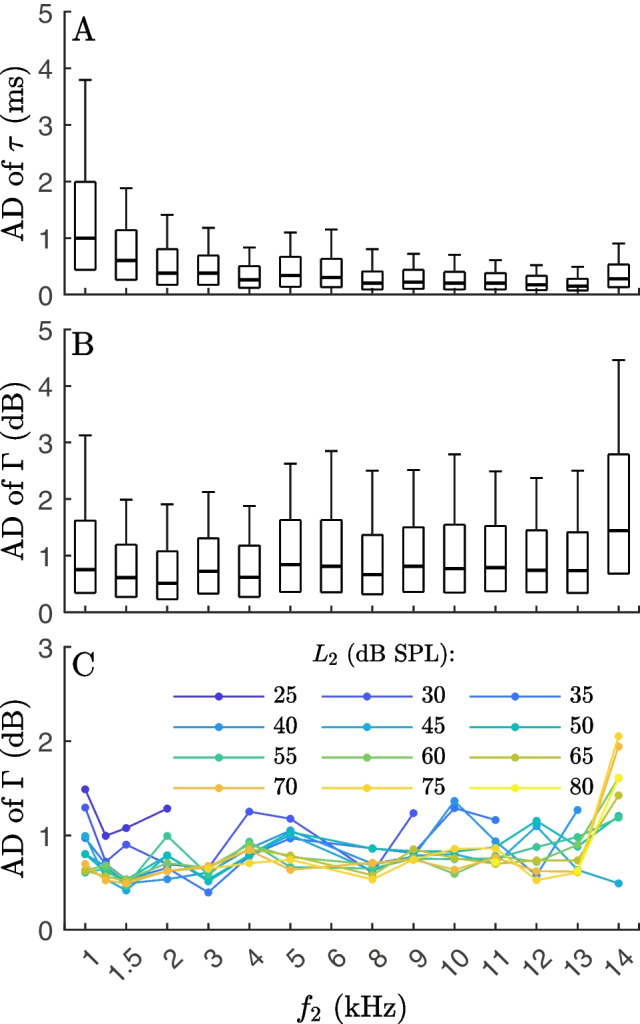
The test-retest reliability of the nonlinear-distortion component latency, $\tau$, presented as absolute differences (ADs) collated across test session combinations (*n* = 21) for each frequency $f_2$. **A** $\tau$ in ms. **B** $\Gamma$ in dB representing the corresponding number of periods in their dimensionless form *N* scaled in dB. One boxplot per frequency. Boxes: IQR from the first to the third quartile. Error bars: 90th percentile of the data. At low frequencies $f_2$ = 1.5–4 kHz, the nonlinear-distortion component latency exhibits high test-retest reliability indicated by low ADs. With the exception of 14 kHz, the median of the ADs of the latencies is less than 0.9 dB, corresponding to a change of less than 10% with minimal variation across frequency. **C** The test-retest reliability of the nonlinear-distortion component latency ($\Gamma$ in dB) presented as the median of AD for every single $f_2$ and $L_2$

Figure 6 shows the test-retest reliability of short-pulse DPOAE latencies of the nonlinear-distortion component, $\tau$, and the corresponding number of periods *N* in their logarithmic representation $\Gamma$ for every single $f_2$ presented as boxplots of their absolute differences (ADs). Interquartile ranges (IQRs) span 1.55 ms at 1 kHz, reducing to 0.26 ms at 12 and to 0.21 ms at 13 kHz. Scaled in dB periods, the ADs show only a moderate frequency dependence. For the frequency range of 1 to 14 kHz, the median is 0.73 dB, and the IQR is 1.12 dB (Table 2). The median for 1–14 kHz corresponds to a test-retest accuracy of 1.41 periods (Table 2). Besides on $f_2$, the test-retest reliability of short-pulse DPOAE latencies $\Gamma$ in dB depends on stimulus level (Fig. 6C, Table Suppl.). At low stimulus levels ($L_2$ = 25–40 dB), $\Gamma$ exhibits lower test-retest reliability than at stimulus levels above 40 dB SPL.

**Table 2:** Test-retest reliability of short-pulse DPOAE latencies of the nonlinear-distortion component extracted with onset decomposition ($\tau$ in ms), the corresponding number of periods in their dimensionless form (*N*) and the number of periods scaled in dB re *N* ($\Gamma$) for each $f_2$

Frequency		AD Median			AD IQR			AD 90%		n
(kHz)								range		
	$\tau$ (ms)	*N*	$\Gamma$ (dB)	$\tau$ (ms)	*N*	$\Gamma$ (dB)	$\tau$ (ms)	*N*	$\Gamma$ (dB)	n
1	1.00	1.00	0.76	1.55	1.55	1.29	3.80	3.80	3.15	1820
1.5	0.60	0.90	0.61	0.88	1.32	0.92	1.88	2.82	1.99	2707
2	0.39	0.78	0.53	0.63	1.27	0.86	1.42	2.84	1.93	2401
3	0.38	1.14	0.72	0.52	1.56	0.98	1.18	3.54	2.12	2524
4	0.26	1.04	0.62	0.38	1.52	0.91	0.83	3.32	1.88	2667
5	0.34	1.70	0.84	0.53	2.65	1.27	1.11	5.54	2.63	2562
6	0.30	1.80	0.82	0.50	3.00	1.28	1.16	6.96	2.91	2386
8	0.21	1.68	0.70	0.33	2.64	1.09	0.83	6.60	2.77	1904
9	0.23	2.07	0.83	0.36	3.24	1.23	0.78	7.02	2.81	1981
10	0.21	2.10	0.83	0.34	3.40	1.29	0.73	7.30	2.97	2073
11	0.20	2.20	0.82	0.31	3.41	1.27	0.66	7.26	2.76	1716
12	0.17	2.04	0.76	0.26	3.12	1.13	0.56	6.72	2.46	1114
13	0.15	1.95	0.74	0.21	2.73	1.11	0.51	6.68	2.62	1035
14	0.28	3.92	1.44	0.40	5.60	2.17	0.91	12.71	4.51	591
1–14	0.30	1.41	0.73	0.52	2.34	1.12	1.21	5.46	2.54	27,481

The test-retest reliability was ascertained with the median of absolute differences (AD), their interquartile range (IQR), and their 90% range (defined as the 90th percentile). The 90% range of AD may serve as a clinical reference to detect pathologic test-retest differences

**Fig. 7:**
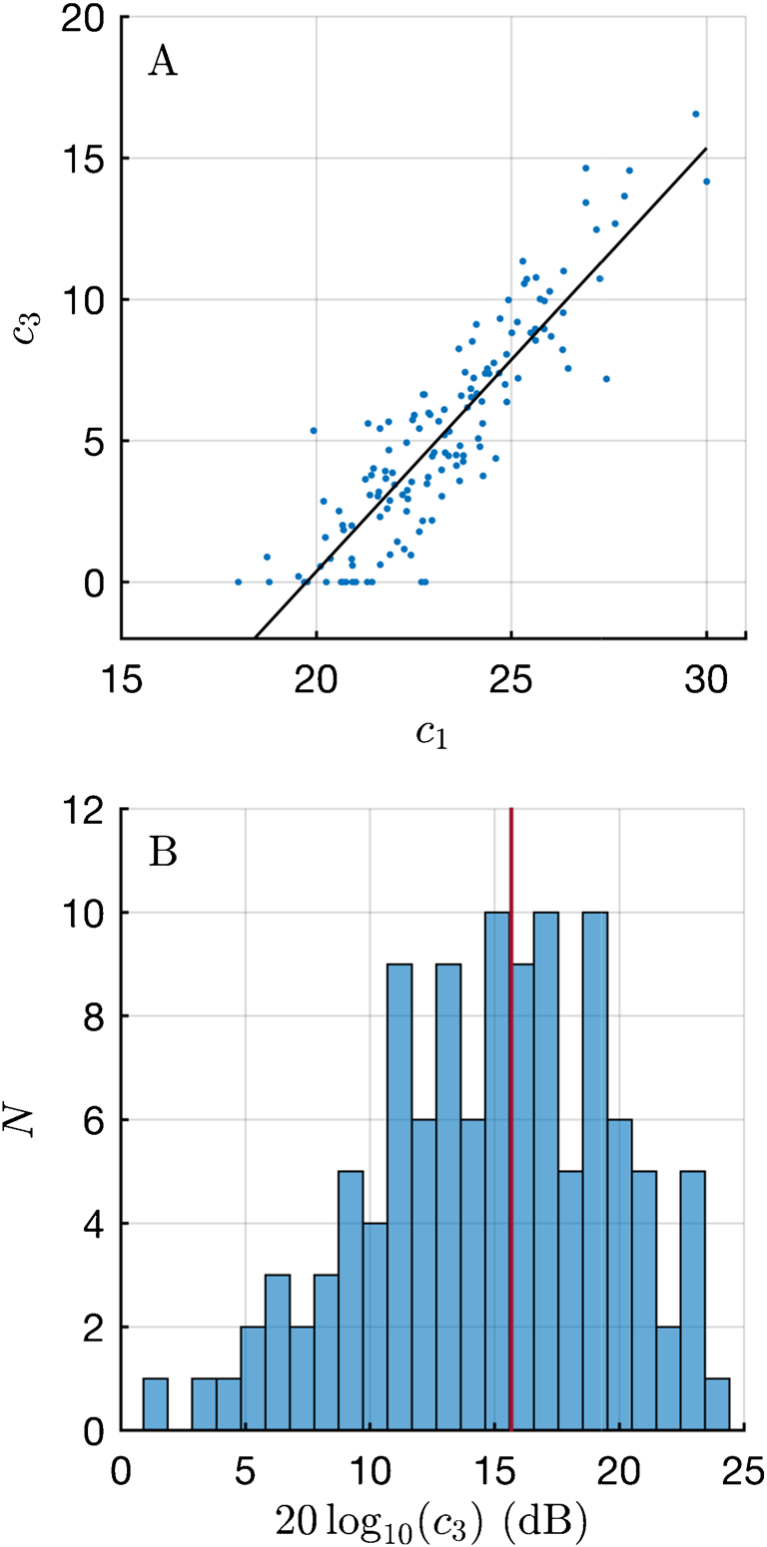
**A** Dependence of the parameter $c_1$, representing the baseline value of the maximum active state of the cochlea, on $c_3$, describing the level dependence of the model function. Linear regression yields $c_3 = -29.5 + 1.497 \, c_1$. This leads to minimum spread of the latencies at 66.7 dB SPL. **B** Distribution of the coefficient of the level dependence, $c_3$, on a logarithmic axis. The vertical red line represents the value of $c_3$ from Table 1 for the linear fit (15.7 dB)

**Fig. 8:**
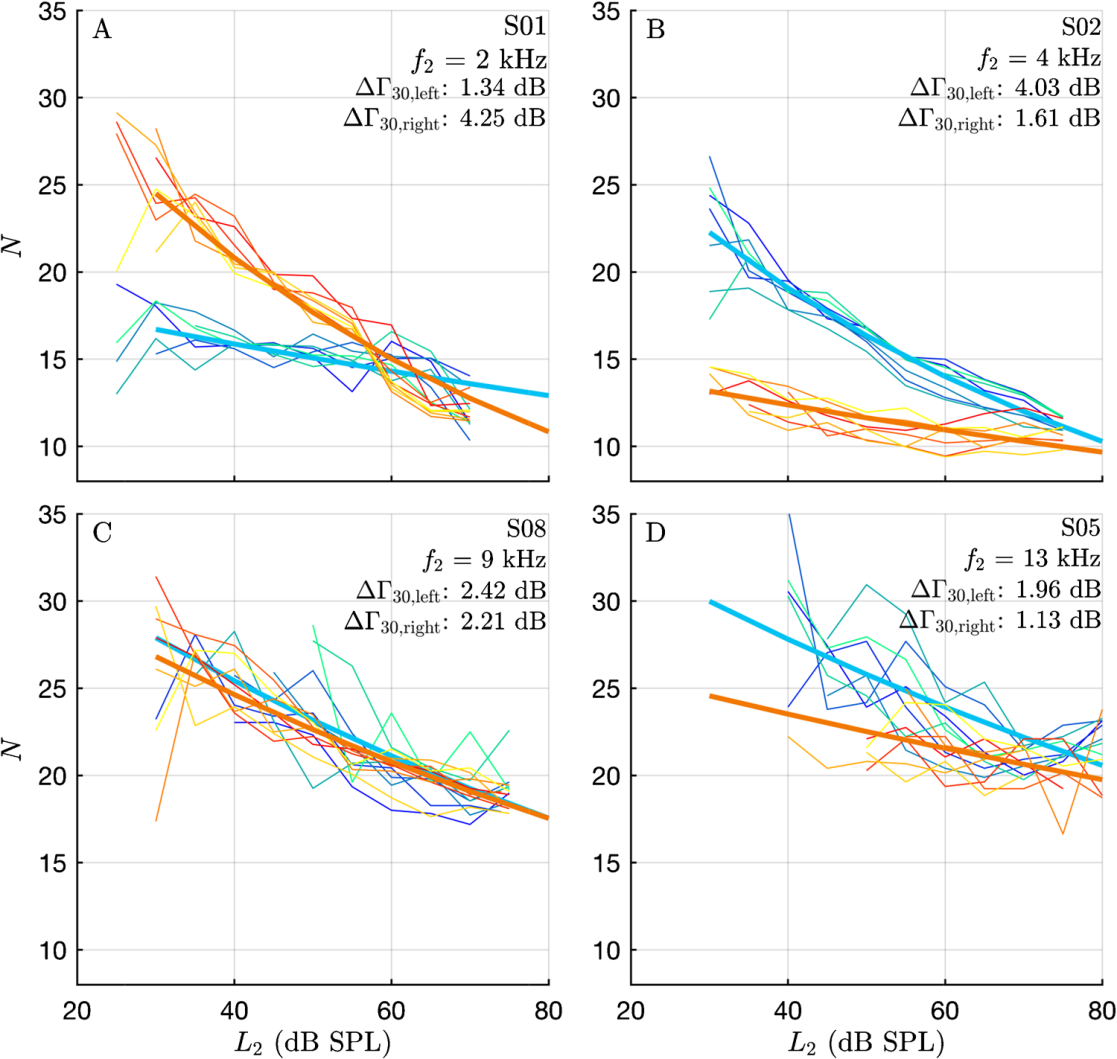
Examples of individual-level dependencies of short-pulsed DPOAE latencies. Each panel represents one subject at a selected $f_2$, each thin line demonstrates one of the seven test sessions, thick lines the curve fits of the level dependence. Blue curves: left ear; red curves: right ear. Inserts show $\Delta \Gamma _{30}$. For instance, in panel **A**, the right ear of subject 01 (orange curve fit) displays a latency of 14 periods at $L_2$ = 65 dB SPL, and 23 periods at 35 dB SPL, corresponding to a change in latency of $\Delta \Gamma _{30}$= 4.25 dB relative to the value at 65 dB SPL (see Methods). At all frequencies shown here, subjects have a threshold of < 10 dB HL with the exception of the right ear of subject S05 at 13 kHz. The examples presented in panels **A** and **B** show exceptions to the general rule: In each subject, at one frequency, one of both ears exhibited a rather low or “flat” level-dependence, where even at the lowest stimulus levels, the latency was not much higher than at 70 dB SPL. Stimulus parameters used for recording the data shown in panels **A** and **B**, and corresponding pure-tone thresholds are given in Table 3. The stability of these measurements over three months indicates that this is not an accidental finding. Panel **C** presents a more typical example, showing a similar level dependence of latencies in both ears. Panel **D** illustrates an example at $f_2$=13 kHz with more scatter, but consistent level dependence of the latencies. Individual ($L_2,\,L_1$)-stimulus level pairs and subjective thresholds $L_{\text {th}}$ in units of dB SPL are given as follows. S01R: ($L_2$=35/$L_1$=46), ($L_2$=65/$L_1$=71); $L_{\text {th}}$=21.5. S01L: (35/49), (65/66); 10.3. S02R (35/62), (65/74); 10.1. S02L: (35/49), (65/68); 20.2. S05R: (35/71), (65/81); 35.1. S05L: (35/68), (65/82); 20.5. S08R: (35/65), (65/76); 11.6. S08L: (35/66), (65/78); 13.8

Figure 7A, shows the mutual dependence of the parameters $c_1$ and $c_3$ describing the level dependence of the model function. Linear regression yields $c_3 = -29.5 + 1.497 c_1$. This regression result, combined with the level-dependent part of Eq. 2, $\tilde{\Gamma } = c_1 + c_3(L_2/100)$, yields a minimum spread of the latencies at $L_2 = 66.8$ dB SPL. When the reference value of 100 dB SPL in the 3rd term of Eq. 2 is replaced by 67 dB SPL, the spread of the parameter $c_1$ is minimized and the dependence between both parameters vanishes (not shown). This indicates that for 67 dB SPL, the inter-subject variation in latency was at its lowest. Figure 7B shows the histogram of $20\,\textrm{log}_{10}(c_3)$, which reveals a smooth, slightly right-skewed uni-modal distribution. A histogram of $c_3$ resembles a negative exponential function or a gamma distribution with $n=1$ (not shown). These findings indicate that the latency properties, as illustrated by the examples in Fig. 8, are part of a continuous distribution. Therefore, these properties should not be interpreted as exceptions, artifacts or as features of a bimodal distribution.

Figure 8 presents four examples of individual level dependencies, highlighting distinct differences between ears. In the upper two panels, deviations from the typical level dependence are observed in one of the ears. For subject S01, at $f_2=2\, \mathrm kHz$, the left ear (blue lines) exhibits a significantly shallower level dependence than the right ear. For subject S02, at $f_2=4\, \mathrm kHz$, the right ear (red lines) shows the shallower level dependence. The bottom left panel presents a typical example where both ears show a similar level dependence for S05 at $f_2=6\,\mathrm kHz$. In the bottom right panel, at $f_2=13\,\mathrm kHz$, the both ears demonstrate the expected level dependence, although with more scatter than in the examples shown in panels A and B. Stimulus parameters used for recording the data shown in Figure 8, and corresponding pure-tone thresholds are given in Table 3.

## Discussion

### General Picture of the Frequency Dependence of Pulsed DPOAE Latency

In this study, the frequency dependence of pulsed DPOAE latency in the range of 1 to 14 kHz roughly follows a power law with an exponent of –0.66...–0.71, where −0.66 is the result of fitting $\Gamma$, and −0.71 is the result of fitting $\tau$. Correspondingly, the frequency-dependence of the periods has an exponent of 0.29 to 0.34, which may be regarded as a proxy for the increase in gain and frequency tuning of the cochlear amplifier observed from the apex to the base of the cochlea.

The range of the above-mentioned numbers reflects different weighting of residuals depending on whether the fit is performed with latency in ms or dB, which is important to consider when comparing to literature values. Figure 5 compares the latency functions from this study to selected data from the literature for a relatively low stimulus level of 40 dB SPL, including SFOAE [41, 42] and DPOAE measurements [41]. For frequencies above 1 kHz, this frequency dependence almost exactly matches the exponent of the frequency dependence for behavioral frequency-tuning in a forward-masking task expressed as tuning-quality factor $Q_{\textrm{erb}} \approx f^{0.27}$, as found in [16] for frequencies of 1–8 kHz (see green curve LOS22 in Fig. 5). The overall trend of these OAE latency measures and their comparison to psychophysical tuning estimates is in accordance with the view that frequency selectivity of auditory neural signals and thus psychophysical performance is basically provided by the frequency selectivity of the cochlear filter, at least at low-to-moderate levels and above 1 kHz. This is consistent with findings from a single preparation in a Chinchilla for a basal location [12], and aligns with the concept that the cochlea and the subsequent neural signal processing provide filtering close to the minimum-phase theorem for linear filtering, as has been proposed for a long time [6].

### Breaks in Cochlear Scaling

The data of this study indicate that the frequency dependence of pulsed DPOAE latency deviates from a single-exponent power law in the frequency band $f_2=3-6\, \mathrm kHz$ (mean value: $c_6=4.7$ kHz). Additionally, there appears to be a noticeable change at $f_2=1.5\, \mathrm kHz$, as the mean pulsed DPOAE latency function, $\Gamma$, shows a higher slope between 1 and 1.5 kHz than between 1.5 kHz and the transition region at 4.5 kHz for seven out of ten stimulus levels (Fig. 3B). The existence of a major basal-apical break in cochlear scaling has been proposed by several authors, typically claimed to be at 1 kHz in humans (for review, see, e.g., [2, 31, 43]). This study, however, included only one frequency below $f_2$=1.5 kHz, so we did not attempt to fit an additional break point.

While earlier studies suggested scale invariance, at least for the basal part of the cochlea, the term “approximate local scaling invariance” [44] is certainly more appropriate and shall be interpreted here as any exponent of the frequency dependence of the periods below 0.3. $2^{0.3}$ = 1.23, meaning that over the range of one octave, properties such as filter bandwidth or latency change “only” by 23%, which might be taken as a reasonable limit for talking of approximate local scaling invariance. In this sense, the region between $f_2=3\!-\!6\,\mathrm kHz$ would appear to clearly violate approximate local scaling symmetry.^2^ This deviation suggests that also in the basal half the cochlea does not adhere to a simple power law scaling across all frequencies but instead exhibits localized variations in frequency tuning and latency properties.

The improved fit of the frequency dependence of latency when adding a tanh-function to fit the transition region, taken together with the high stability of results over three months, suggests that this is not an incidental finding. These deviations from a simple power law differ from those discussed by others. Christensen et al. [33] identified a second break at 2.6 kHz, beyond which the increase in their DPOAE “scaled” phase — a method to reduce the influence of the different frequency ratios they used — diminishes. Their phase, presented as periods on a linear scale, i.e., *N*, differs from $\Gamma$ as used in this study. They also noted a break in the corresponding SFOAE measure, which steepens above that transition frequency. After rescaling the data from [33] to $\Gamma$ (not shown), a segmented linear fit to the SFOAE data would show breaks at 350 Hz (clearly) and at 1.5 kHz (weakly). Overall, their SFOAE curve shows more continuous changes in bending rather than clear breaks.

For their corresponding DPOAE data, the most notable feature of $\Gamma$ is a break at around 4 kHz, above which the periods remain constant. Comparing these findings to log-log scaled mean curves recorded at sound-pressure levels of 40 dB SPL (Fig. 5), we could be tempted to identify various weak breaks in the curves. However, no common feature consistently appears in the range of 1–5 kHz across all curves. Although not firmly evidenced, the comparisons in Fig. 5 suggest that determination of latency in the time domain (this study and curves MS16) shares some features, while curves based on the phase-gradient method show different characteristics (curve AGS18). The salient common feature of OAE data, from which one would expect to be able to infer something about cochlear scaling, is a rather constant rise in periods of around 0.3 dB/dB throughout the range of 1–10 kHz.

Weak breaks or perturbations in the frequency dependencies of latencies appear to exist, and in this study, they might even be said to be pronounced and also clearly consistent across the different stimulus levels. Moreover, these breaks remained individually stable over a three-month measurement period. Their exact shape seems to be quite dependent on subject (Fig. 4C, D), analogous, for example, to the spread of fitted break frequencies of [33]. Similarly, when deriving cochlear properties from swept-tone DPOAE phase gradients, the level-dependence is very low [45], while, in contrast, recent studies on SFOAE show clear level-dependence throughout the frequency range of 0.7–8 kHz [46], aligning fairly well with this study for low-to-moderate levels.

To conclude on the scaling break issue, it is conspicuous that the PTC of Oxenham & Shera (curve LOS22 in Fig. 5) also shows a steepening of the slope above 4 kHz, although no “saturation” indicates the end of a transition region. The forward-masking PTC (1–8 kHz) had been measured at 10 dB SL [16]. As threshold pressure at the eardrum rises by 5 to 10 dB from 1 to 8 kHz (cf. Figure 3 in [47]), the PTC data have probably been measured at 15 to 25 dB SPL. When correcting for higher sound pressures at higher frequencies, the level dependence for a fixed stimulus pressure suggests that the $Q_{\textrm{erb}}$-values would rise, leading to a better match to the pulsed DPOAE latency frequency dependence. These differences highlight the complexity of directly comparing results across studies and underline the need for cautious interpretation of scaling breaks in cochlear measurements.

### Comparison of Pulsed DPOAE Latencies to ABR Latencies

Pulsed DPOAE latencies are compared to those of ABR using data from tone-burst evoked ABR wave V [27] and from click evoked electrocochleographic NAP wave I measurements [7]. While the frequency-dependence of the NAP wave I data matches the general 0.3 dB/dB dependence seen in all OAE data in Fig. 5, the tone-burst ABR wave V data do not. The study of [27] extended that of [26] by varying ramp designs. The data shown belong to tone-burst durations scaled with $f^{-0.5}$, covering a frequency range of 1–8 kHz. To facilitate comparison, twice the estimate for cochlear forward latency is presented, calculated by subtracting 5 ms from the wave V latency [27]. This adjustment accounts for a 1 ms synaptic delay and a 4 ms interpeak wave I-V latency [26, 27]. These data were excellently reproducing the data of [26].

**Table 3:** Stimulus-related parameters and individual pure-tone thresholds for panels A and B of Fig. 8

	S01 le	S01 ri	S02 le	S02 ri
$L_1$ @ 35	49	46	49	62
$L_1$ @ 65	66	71	68	74
Def. of $L_1$	IL	IL	IL	PL
*a*	0.57	0.83	0.63	0.40
*b*	29.1	17.0	27.0	48.0
$R^2$	0.96	0.99	0.98	0.99
$L_{\text {th}}$	6.0±1.9	−5.1±2.1	5.1±2.8	0.8±4.4

$L_1$ is given in dB SPL for $L_2=35$ and 65 dB SPL. Definition of the stimulus path was either individually derived from an independent level-map measurement (IL) or from population mean parameters (PL). $a,\, b$: parameters of the stimulus path according to $L_1 = aL_2+b$. $R^2$ is the squared correlation coefficient of the model fit to the level map from which the individual path was derived. $L_{\text {th}}$ is the mean Békésy threshold across the seven visits given in dB HL

Two curves are shown (RAN13, gray curve, and RAN13 corr., red curve), where the latter includes a correction to estimate the correspondent group delay, because the data given in both papers compute the delay from stimulus onset, as is common usage in audiology. However, in the case of tone-burst stimuli, the group delay is the shift between stimulus and response pattern. Therefore, twice the ramp duration from the round-trip latency was subtracted. The derived round-trip group delay latency aligns reasonably well between 2 and 4 kHz with the OAE latencies shown in Fig. 5 but diverge notably at higher frequencies. At 8 kHz, the highest frequency of the data of [27], the discrepancy at 40 dB SPL amounts to a factor of 1.6 (37.3 vs. 22.8 periods, Fig. 5) or additional 1.8 ms for the round-trip latency, which is considerable. In the study of [27], ABR latency was also compared to tone-burst OAE (TBOAE). A slightly disproportionate increase of ABR versus TBOAE latency had been noted by [27] themselves, who, using the same stimulus waveforms, measured tone-burst OAE extracted by the nonlinear-residual technique, and derived the energy-weighted group delay as their measure of latency. At 8 kHz and the stimulus level of 40 dB SPL, their wave-V delay was 8.2 ms, resulting in a forward delay estimate of 3.2 ms, and the TBOAE delay was found to be 5 ms, resulting in a forward delay estimate of 2.5 ms. Consequently, the ABR data overestimates the TBOAE latency data if a factor of two is used to convert OAE round-trip delay to ABR forward latency. Their TBOAE latency of 5 ms is also considerably longer than the DPOAE latency of this study (2.8 ms). The authors discuss the reason for overestimating tone-burst OAE latencies, especially at high frequencies and low stimulus levels, i.e., the necessity to resolve the nonlinear residual in an increasingly noisy portion of the settling stimulus signal. A major difference compared to the data of this study lies in the pulse widths used for stimulation; for example, at 1 kHz, the full width half maximum of our $f_2$ pulse was 13.1 ms, whereas it was 1.6 ms in the study of [27].^3^

Latencies of wave I derived by transtympanic electrocochleography, stimulated by clicks with appropriate high-pass noise masking of the basal emitters [7], however, intended to limit the region contributing to the narrow-band electrocochleographic NAP to ½ octave basal to the characteristic frequency place (CF; 3-dB criterion). The latencies were fitted with $\tau = \tau _0 + 3.4 f^{-0.77}$, *f* = 0.45–10 kHz, where $\tau _0$ = 0.8 ms accounts for the synaptic delay. The exponent of −0.77 is in close agreement with the OAE data shown in Fig. 5. At 10 kHz, the computed latency of 11.6 periods is much lower than in the data of [26] and [27]. Here, a transition region between the data points at 3.6 and 5.3 kHz can be clearly identified.

**Fig. 9:**
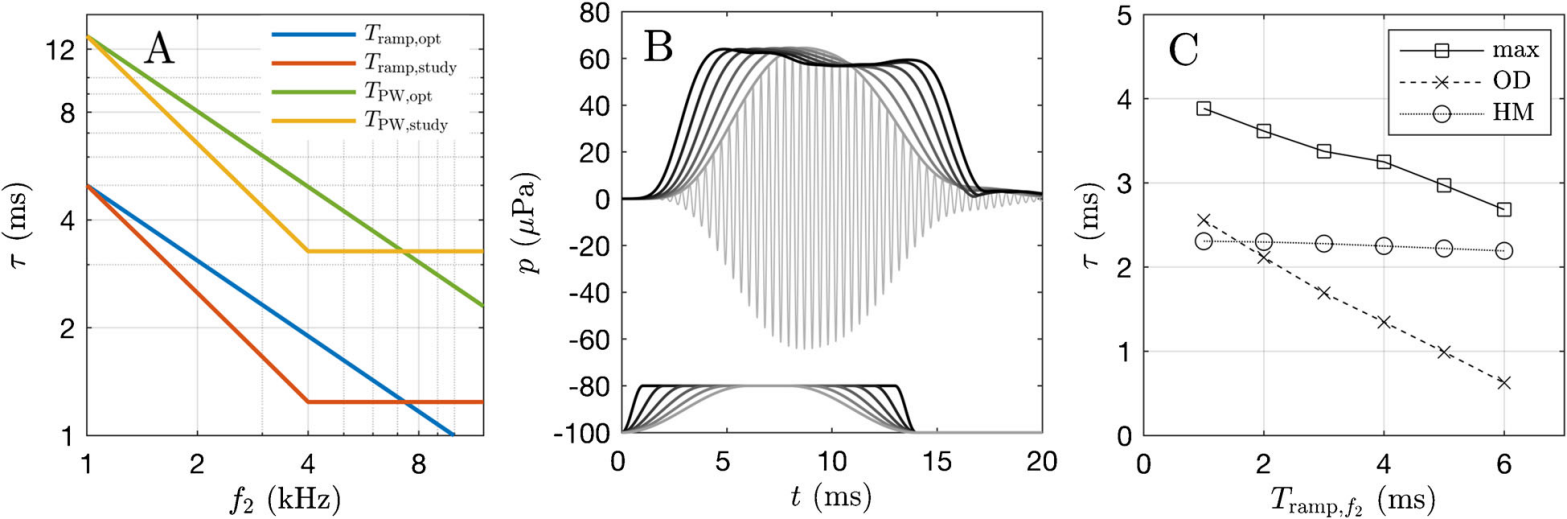
Ramp design and influence on time-domain measures. **A** Frequency dependence of ramp duration ($T_\textrm{ramp}$) and full width half maximum ($T_\textrm{PW}$) of the $f_2$ short pulse, shown for the post-hoc optimal design (blue/green line), and as used in this study (yellow/red line). **B** Time course of a DPOAE pulse response, as computed with a nonlinear active model of the cochlea [20, 52], for six different ramp durations $T_{\textrm{ramp,}f_2}$=1–6 ms at $f_2$=4 kHz. **C** Dependence of the latency, according to the definition used of our experimental results, i.e., $t_{\textrm{OD}}-t_{\textrm{ss,}f_2}$ (OD), on ramp duration. In addition, latency is computed for $t_{\textrm{max}}-t_{\textrm{ss,}f_2}$ (max) and half-maximum values for ramp and pulse response (HM). As ramps at 4 kHz to be exaggeratedly shorted by 0.73 ms, the resulting bias due to ramp design corresponds to an additional lag of 0.20 ms or 0.8 cycles and thus does not explain the relatively shorter latency at the beginning of the transition region

Level-dependence also allows a glimpse on what might contribute to the discrepancy between ABR latencies of Neely and Rasetswhane [26, 27] as compared to the OAE literature. ABR latencies are dominated by the most basal regions of the cochlea, where the inner hair cells first reach the threshold of synaptic firing during the build-up of a tone burst. As the stimulus levels increase, the earliest generators move faster towards the base, i.e., move from a tail-side $Q_{\textrm{10dB}}$ point at low stimulus levels successively to, say, the $Q_{\textrm{40dB}}$ point at a higher level, which is expected to lead to a stronger dependence of latency on stimulus level. In the present study, over a range of 45 dB level variation, the latencies vary by 2.8 dB (Fig. 4A), whereas in the ABR data of [26] and [27] the correspondent change is 6.3 and 6.5 dB, respectively. This notion would imply that frequency-specificity of tone-burst ABR is more reduced with higher stimulus levels than the OAE generation region of the nonlinear-distortion component, and this in turn could provide a plausible contributor for the discrepancy in the exponent of the latency dependence as well. However, such concepts are based on steady-state properties such as tuning quality factors, and consequently should be preferably investigated as a transient process in a time-domain model.

### Test-Retest Reliability of Pulsed DPOAE Latencies

The test-retest reliability of pulsed DPOAE latencies might become clinically relevant, in combination with short-pulse DPOAE amplitudes or eventually as a stand-alone measure, for instance, to objectively monitor the function of the cochlear amplifier. To provide a reference range in ten normal-hearing subjects, the test-retest reliability of nonlinear-distortion component latencies $\tau$ was determined by repeatedly testing the subjects seven times over three months (Fig. 6, Table Suppl.).

There are only a few reports on the test-retest reliability of DPOAE latencies. Mahoney and Kemp [48] reported that the test-retest reliability of DPOAE delays was within 8.5% of the mean at $f_2$ = 1–6 kHz tested in 12 ears three times by using an $f_2$ ratio sweep with the $f_2/f_1$ ratio 1.22–1.26, $L_1$/$L_2$ = 60/45. In the present study, test-retest comparisons of $\Gamma$ (dB re *N*) were within 6.0% of the mean for $f_2$ = 2 kHz and within 9.1% for $f_2$ = 6 kHz. Dreisbach et al. [49] described the test-retest reliability of DPOAE delays in normal-hearing adults for $f_2$ = 2–16 kHz using $f_1$ ratio sweeps with a fixed $f_2$ and varying $f_1$, resulting in frequency ratios of $f_2/f_1$ = 1.05–1.30 and $L_1$/$L_2$ = 60/45. The average group delay differences were 0.28 ms (SD 0.24 ms) at $f_2$ = 2–8 kHz and 0.22 ms (SD 0.20 ms) at $f_2$ = 10–16 kHz compared with the present study with 0.29 ms (SD 0.54 ms) at $f_2$ = 2–8 kHz and 0.21 ms (SD 0.31 ms) at $f_2$ = 10–14 kHz.

Dreisbach et al. [50] measured DPOAE ratio sweeps four times in 40 cystic fibrosis patients at the two highest frequencies where patients had present DPOAEs. The average absolute difference between trials for group delay at $f_2$ = 8–16 kHz was 0.23 ms (SD 0.33 ms), with the smallest absolute differences of 0.19 ms occurring at $f_2$ =14 kHz and the greatest absolute differences of 0.29 ms at 16 kHz. In the present study, the smallest absolute difference occurred at $f_2$ = 13 kHz with 0.17 ms, and the greatest absolute difference occurred at $f_2$ = 14 kHz with 0.28 ms within $f_2$ = 8–14 kHz. The 95% range of data amounted to 0.87 ms at $f_2$ = 8–16 kHz [50] in comparison with the present study with 0.93 ms at $f_2$ = 8–14 kHz. In summary, the test-retest reliability of short-pulse DPOAE latencies described in this study seems to be comparable with the test-retest reliability of DPOAE group delays using $f_1$ ratio sweep and $f_2$ ratio sweep paradigms based on the literature.

The observation that the absolute differences of $\Gamma$ show basically only a weak frequency dependence (see Fig. 6B and Table 2), with the median ranging between 0.53 and 0.84 dB for 1–13 kHz, illustrates that the intra-subject reliability, test-retest reliability or stability of the latency is not dominated by noise or systematic properties of the OD algorithm. If it were, then the test-retest reliability would be expected to be primarily constant in terms of absolute differences of $\tau$, where, however, the correspondent range of medians spans between 0.15 and 1.0 ms. This observation suggests a physiologic cause for latency stability.

### Possible Influence of Ramp Durations and Pulse-Width Choice

The question may arise as to whether the transition region observed between 3 and 6 kHz in our data could be attributed to our ramp duration choice. Figure 9A illustrates the ramp durations ($T_\textrm{ramp}$) and pulse widths ($T_\textrm{PW}$) that would be deemed optimal post-hoc, i.e., following $\tau \approx f^{-0.7}$, along with those chosen in our experiments. The divergence between the functions can be expressed such that our experimental $f^{-1}$-dependence overcompensates cochlear dispersion by up to a factor of 1.53 (=1.93 ms/1.26 ms) up to 4 kHz, and then reduces the overcompensation due to the constant ramp duration above 4 kHz, reaching parity with the optimal choice at 7 kHz.

Our latency definition counts the time elapsed between the stimulus reaching steady state and $\tau _{\textrm{OD}}$. In the mean, amplitude measured at $\tau _{\textrm{OD}}$ is 1.67 dB below the maximum (or steady state) of the pulse response, i.e., at 83% [51] (see also the example shown in Fig. 2). We now consider two possible scenarios.

#### Scenario 1

The nonlinear, active amplification in the cochlea responds with a delay, but can ideally follow the ramp form. In a cosine-law, 83% are reached 0.81 = 1–0.19 of the ramp-up time. Therefore, 19% of the difference between the ideal $f^{-0.7}$ ramp definition and the experimental one is expected to confound our results, i.e., 0.19   (2.14–1.41  ms) = 0.14  ms, corresponding to approximately half a period at 4 kHz. This is expected to lead to an artefactual reduction of the latencies reaching its maximal amount at 4 kHz, thus potentially explaining the transition region, but not to the amount seen in the experiments. First, Eq. 2 leads to a relative latency reduction of 0.29 ms for the $c_{1...6}$ parameter set at 4 kHz; thus, only 45% of the latency discrepancy between both model fits at 4 kHz could be explained. Second, the steepness of the latency increase in the transition region is much less explainable by the ramp choice: Between 4 and 6 kHz, the discrepancy between both ramp designs amounts to 2.76 cycles, which at the mean frequency of 5 kHz corresponds to 0.55 ms. However, only 19% of this — equivalent to 0.4 cycle at 5 kHz — is expected to bias our latency measure. This does not explain the transition region, and moreover, the question would arise why there is the second bend at the end of the transition region.

#### Scenario 2

For instance, we assume that at 1 kHz the cochlea can follow the ramp, but as the ramps become exaggeratedly short up to 4 kHz, the cochlea can no longer follow the transient quick enough, due to some sort of slew-rate problem. In this case, the delay would be relatively prolonged by maximally (2.14–1.41  ms) = 0.73  ms at 4 kHz, i.e., 2.9 cycles. Thus, it appears that a slew-rate problem could certainly lead to considerable effects, but it would explain at first sight a relative increase of the latencies up to $f_2$=4 kHz, followed by a decrease up to 7 kHz and higher.

To further test a possible ramp-design influence, we simulated the influence on latency measures using a one-dimensional hydromechanical nonlinear cochlea model solved in the time domain [20, 52]. This model type replicates the short-wave behavior close to the peak [64], taking into account that pressure variation in the scalae may narrow down to a region in the vicinity of the basilar membrane, a phenomenon also called fluid focusing [53]. The model is coupled to a multi-component oscillator system mimicking realistic middle-ear transmission properties. Six different ramp durations were tested at $f_2$=1.5 and 4 kHz. Figure 9B shows exemplarily the DPOAE pulse responses in the ear canal obtained for $f_2$=4 kHz. Inspection of Fig. 9B reveals a slew-rate-like phenomenon, because it is clearly seen that for ramps shorter than 4 ms, the DPOAE pulse response increasingly fails to follow the onset with high fidelity.

Figure 9C depicts the dependence of latency on ramp duration for three different latency definitions. For this discussion, the most important is the latency computed as the time elapsed between the end of the stimulus waveform ramp and the OD point of the DPOAE pulse response (Fig. 9C, dashed line with crosses), similar to the experiments discussed here. At 4 kHz, where the ramp duration used in our study was 1.41 ms, whereas 2.14 ms would have been optimal (Fig. 9A), the correspondent change in measured latency due to this non-optimal choice corresponds to a potential exaggeration of the latency of 0.20 ms. In contrary to our results, this means that ramp duration choice would have led to a latency value exaggerated by 0.8 cycles at 4 kHz and an understated estimate for higher frequencies. Thus, following the model, the transition region would even be understated. Note that this type of cochlea model represents not a quasi-linear approximation of a nonlinear system, but solves the transient response of a nonlinear distributed positive-feedback system in the time domain.

To conclude on this issue, a minor influence of ramp duration choice on the transition region cannot be excluded, but is not expected, given that (1) the combination of ramp design and the definition of the latency using the onset-decomposition algorithm predicts a clearly smaller effect, (2) an explanation involving a slew-rate problem leads to an opposite effect, and (3) the nonlinear, active model predicts a dominance of the slew-rate effect. While a general existence of such a transition region in normal-hearing subjects is questionable, the above considerations and the simulations show that ramp design hardly contributes considerably to the finding that the individual latency functions can show clear transition regions (Fig. 4C, D) that differ inter-individually in amount and position, and are partially so steep that the assumption of scaling symmetry in the above-mentioned sense becomes unrealistic in certain frequency regions.

Another limitation could be seen in using pulse widths that are clearly larger than the expected latency at frequencies above approximately 8 kHz, at least for higher stimulus levels. For $f_2\ge 4$ kHz, the full width half maximum of the pulses is 3.2 ms, which corresponds to about 24 periods at 8 kHz and already 30 periods at 10 kHz. The question arises whether a contamination by the coherent-reflection source could skew the latency data. For instance, Fig. 1B from [35] shows a pulse-basis decomposition of a pulse response at 10 kHz. In that example of a high-frequency pulse response, there appears to be a smaller coherent-reflection source with a delay relative to the nonlinear-distortion response of $\approx$2.5 ms and a clearly different phase (almost in quadrature). Although the stimulus pulse width is approximately twice the delay between both source contributions, the coherent-reflection source just starts at the onset decomposition time of the sampling algorithm, and thus does not interfere with it. At frequencies higher than 10 kHz, there might have been a risk of falsely sampling an interference state between two sources. On the other side, we have never encountered amplitudes of a presumed coherent-reflection source at such high frequencies being much larger than the one shown in Fig. 1B from [35]. One has to keep in mind that using the time-domain method, once in a while one would encounter a destructive phase constellation which, if both contributions have similar amplitude, always would strike the eye, presenting a notch where both response contributions overlap, which we never saw. It is thus deemed improbable that the latency data, even up to 14 kHz, are contaminated to any great extent by interference phenomena between both source contributions.

### The Factor of 2 and Whether OAE Are Backpropagated by Compressional or Slowly Traveling Waves

We have used here a factor of 2 to make ABR forward delay comparable to the raw latencies of our and others’ OAE data, as well as those reported by others. This is a choice which we have borrowed from comparisons of tone-burst (TBOAE) and click-evoked otoacoustic emission (TEOAE) latencies to ABR data in the past [26–28, 54]. For instance, Rasetshwane et al. [27] found that TBOAE latency was 2.23 times ABR forward latency, for $1.5\le f \le 8$ kHz and a stimulus design following an $f^{-0.5}$ law. Harte et al. [28] reported a factor of 1.92, and Moleti and Sisto, using click-evoked OAE (TEOAE), and including data of two earlier studies, found a factor of 2.08 [54]. SFOAE have been shown to exhibit frequency dependence and emission latency nearly equivalent to TEOAE [55]. Taken together, TEOAE, when analyzed in the frequency domain, TBOAE, and SFOAE appear to be dominated by the coherent-reflection mechanism generating an emission response at the place of the traveling wave peak, and thus, comparing their emission delay to the forward delay of ABR responses equivocally supports the assumption that OAE backpropagation requires approximately the same amount of time than the forward propagation. While comparison between ABR and OAE latencies is critically dependent on calibration of stimuli, ramp definitions used, level-dependent basal contributions to ABR signals, amount of inter-subject variability, to name just a few, the majority of these above-mentioned experiments in humans support a factor close to 2.

Before addressing the slow vs. fast or compressional wave dispute, we try to lay out a hand-waving version of the slow-wave hypothesis for DPOAE, which, contrary to the case of TEOAE, TBOAE, and SFOAE, has to take into account the different frequencies in play.

According to classical 4-terminal network system theory, a passive, linear transmission line is a reciprocal system. This general property has been recently demonstrated for the case of cochlear mechanics (see appendix in [65]). For the case of DPOAE, the hand-waving argument would run as follows: A traveling wave with $f_2$ reaches its characteristic place after a certain forward delay, $\tau _f$. At this place, the outer hair cells induce actively pressure having the new frequency component $f_\textrm{DP}$ into the cochlear fluids. The reciprocity property then demands that the time for the wave to travel back corresponds to the time that a forward-traveling wave of this frequency would take to reach this place, being tonotopic for $f_2$ and not $f_\textrm{DP}$. Now inspecting measurements of basilar membrane movement in live animals and gauging travel time toward a place at $f_2/f_\textrm{DP}=2/3$ basal to its tonotopic place with the derivative of phase curves e.g from Fig. 1 of [66], the backward travel time would correspond to $\approx$1/6 of the forward travel time, reflecting the fact that most of the latency accumulates in the vicinity of the peak. Following this argument, we would now set the factor relating DPOAE travel time to pure forward delay of the $f_2$ wave to 1.17 or even closer to 1, probably indistinguishable from a compressional wave. Such a simple argument based on application of the reciprocity theorem neglects three problems: (1) the active, nonlinear properties of the cochlea violate the theorem’s requirements, (2) the force production is distributed [66], and (3) even if the reciprocity theorem would be approximately valid, it requires that one reproduces force *and* velocity (or current and voltage) at the far end, which poses a requirement to the load for the retrograde transmission case. Thus, estimation of the expected retrograde delay in the case of the slow-wave assumption is non-trivial.

The dominance of a slow backward traveling wave for OAE generation has been questioned, due to the absence of the expected phase gradients in a living cochlea [68, 69]. However, the inability to prove a retrograde traveling wave does not prove its absence. Simulations of cochlear models support the dominance of slow backpropagation and offer explanations for the failure to detect the expected phase gradients in basilar membrane experiments [65, 67].

**Fig. 10:**
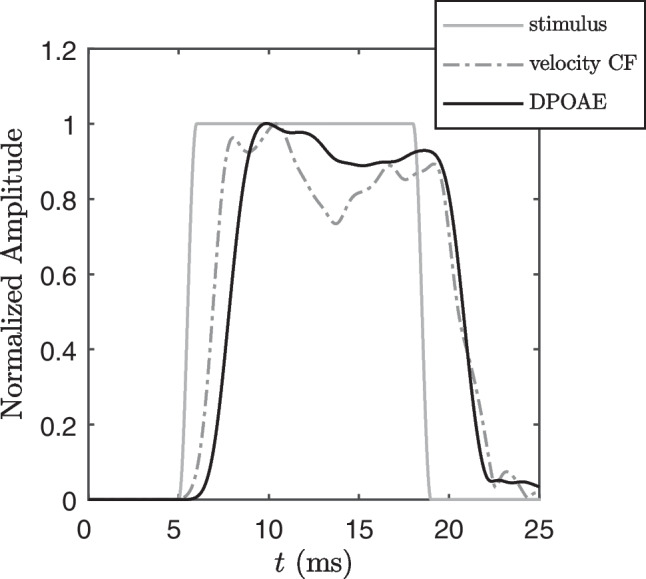
Simulation of the response of the cochlear partition at the tonotopic $f_2$ place filtered at the DPOAE frequency $f_\textrm{DP}$ (“velocity CF”), and the ear-canal DPOAE pulse response (“DPOAE”), along with the $f_2$ stimulus waveform (“stimulus”). Clearly, the ear-canal DPOAE waveform lags the cochlear vibration response at the generation place of the nonlinear-distortion component of the DPOAE, although the backpropagation time amounts only to 65% of the forward propagation time of the stimulus (shown for $T_{\textrm{ramp}}$=1 ms)

In the context of this study, it is important to note the discrepancy between ABR and TBOAE (also our DPOAE) latencies, if one would assume a factor close to 1 (negligible slow wave traveling back): At 2 kHz and a stimulus pressure of 40 dB SPL, the ABR wave V latency was found to be 11 ms, whereas the TBOAE latency was 8.7 ms (Rasetshwane et al., 2013). If the latter reflects only the cochlear forward delay (compression wave hypothesis), only 2.3 ms would remain for synaptic delay and wave I-V inter-peak delay. Considering that inter-peak latency is 4 ms, and synaptic latency has never been seen to be below 0.8 ms, the discrepancy to be reconciled is 2.5 ms. Taking into account that 40 dB SPL for a single tone burst of 2.8 ms duration is close to threshold (about 30 dB SPL), substantial basal contributions to nerve spikes leading to an artificially shortened latency are not a plausible explanation for this discrepancy.

Ultimately, we simulated a short-pulse DPOAE stimulation in a nonlinear, active cochlea model [20, 52]. Figure 10 shows the pulse response of the cochlea at the tonotopic place of $f_2$, filtered at $f_\textrm{DP}$, and the ear-canal DPOAE pulse response along with the $f_2$ short pulse eliciting the DPOAE, for $f_2$=4 kHz. The forward propagation of the $f_2$ pulse takes 1.4 ms, while the backpropagation of the generated distortion product takes 0.91 ms (all measures taken at full width half maximum). This would correspond to a factor of 1.65, which should be understood as an estimation of the lower boundary for the share of the forward delay, because OHCs situated within ½ octave basal to CF are expected to contribute to the $2f_1-f_2$ pressure induced by the OHCs into the fluids. The factor should depend on the frequency ratio, and it is also expected to depend on the level ratio because of the influence of mutual suppression of both waves. In this respect, a strength of this study is its ability to derive latency from DPOAE measurements, where the level combinations correspond to an individually, frequency-specific optimal path.

In the comparison of OAE latencies to ABR latencies shown in Fig. 5, using the factor 1.65 instead of 2 would shift the ABR forward latency curve of [27] downward, placing it a slightly below our DPOAE values in the frequency range of 2–6 kHz. If we can rely on our model, we would have to divide the one-frequency OAE latency data by 2/1.65=1.21 to compare to our DPOAE, leading to slightly smaller values than ours. Factors below 1.5 or below 2 for the TEOAE, TBOAE or SFOAE measurements shown in Fig. 5 would lead to discrepancies between OAE and ABR forward latency estimations, which would become increasingly challenging to reconcile.

The simulations provide support for the view that slow-wave propagation dominates the DPOAE backpropagation mechanism. The calculated delay ratio, the close correspondence of the correspondent forward delay with other OAE latency results (Fig. 5), and the relation to ABR forward latency estimates support the validity of slow-wave assumptions in explaining OAE backward propagation. Nonetheless, the compressional vs. slow-wave dispute is certainly not settled here.

### Comparability of Cochlea Latency Estimations From Nonlinear-Distortion-Source DPOAE vs. SFOAE

Over the past decade, research on cochlear latencies and their relation to OAE has leaned towards SFOAE. This trend may be attributed to the seemingly greater complexity involved in the analysis of DPOAE and the methods to acquire them. For instance, the swept-primary DPOAE method with derivation of the latency using the PGM introduces three main complexities into cochlea latency estimation: (1) Two waves of different frequency are required to evoke a DPOAE, (2) the retrograde wave occurs at a third, different frequency, (3) the PGM produces a delay that to the first approximation, i.e., in a scaling-invariant cochlea, and assuming a slow-wave interpretation, represents the sum of the forward travel time of the distortion-product ($f_\textrm{DP}$) from the $f_2$-place to its characteristic place plus the delay of the coherently reflected wave back to the stapes. In this study, complexity (3) is removed, but the first two remain. These may be alternatively described as “mutual suppression complexity” of the primaries and the second, the “wrong place — different frequency complexity” in the $f_\textrm{DP}$ retrograde wave that is initiated at the characteristic place of $f_2$ (recall that $f_1$ plays no role with respect to latency in the $f_2$-short-pulsed paradigm used here). In contrast, an analysis of SFOAE latencies might appear much more straightforward, because forward and retrograde latency is produced by a wave of the same frequency, traveling across the same path.

In the protocol used here, the level difference $L_1-L_2$ is individually adjusted to yield the maximum response pressure $p_\textrm{DP}$. This approach ensures optimal control of suppression effects, improving consistency across subjects and frequency. Conceptually, this adjustment maintains similar intra-cochlear amplitudes of both traveling waves within the overlap region, probably extending roughly ½ octave basal to the characteristic place of $f_2$. This can be said because if one were to trade the one wave for the other while keeping the sum of both constant, in the two limiting cases ($L_i \rightarrow 0$), no distortion product could be produced.

Suppression is expected to lead to a gain reduction as compared to a non-suppressed state, and thus to a reduction in latency as well. However, comparison of SFOAE (“MS16” and “AGS18”) and nonlinear-distortion source DPOAE (“Pulsed DPOAE”) latencies ($L=L_2=$40 dB SPL) shows that the latter is equal or even slightly higher (Fig. 5). Several factors may explain this observation: (1) the degree of suppression is not high enough to have a considerable effect, (2) the suppression effect is balanced by a smaller reverse-to-forward latency ratio for DPOAE than for SFOAE, (3) the suppression effect is balanced by a smaller effective region contributing to DPOAE than to SFOAE, or any combination of (2) and (3). For instance, assuming the OAE-to-forward delay ratios discussed in the “The Factor of 2 and Whether OAE Are Backpropagated by Compressional or Slowly Traveling Waves” section, and the OAE latencies from Fig. 5 at 3 kHz (where they are approximately equal at 16 periods), hypothesis (2) implies the following: Given a reduced OAE-to-forward delay ratio of 1.65 in case of the DPOAE, the forward delay would be 9.7 periods, whereas for the SFOAE, the forward delay is 8 periods (taking the summary value of 2 from the “The Factor of 2 and Whether OAE Are Backpropagated by Compressional or Slowly Traveling Waves” section). Recall that the factor 1.65 for the DPOAE includes the suppression effect by nature of the underlying model. Therefore, to reconcile both numbers, we would need to add hypothesis (3) and conclude that the effectively contributing region for the SFOAE was somewhat larger than in the case of DPOAE, in order to compensate for the finding of a shorter forward latency. This is, of course, just a numbers game. For instance, reports on animal experiments have shown that the SFOAE-to-basilar-membrane latency ratio is actually 1.6–1.8, thus considerably below 2, and a physical explanation based on a model has been presented [56, 57]. Nonetheless, we might conclude that these considerations do not indicate that mutual suppression has a dramatic effect on the latency estimates derived from the short-pulse DPOAE measurements presented here (at least, for low-to-moderate levels and for the mid-basal frequency region in humans).

However, the relationship between cochlear and OAE latencies bears also complexities that are exclusive to SFOAE. Depending on frequency, level, species, and individual condition, these might comprise nonlinear coherent reflection, place-fixed reflection contributions in the tail region, or nonlinear interaction of the suppressor used to derive the SFOAE [57]. Also, from a theoretical point of view, it should be noted that the additional process required to explain coherent-reflection OAE, i.e., the micromechanical irregularity, typically chosen as a  1% perturbation of the underlying active impedance does not, in principle, influence the afferent signaling. Therefore, any pathologic variation of the degree, but more importantly, its macro profile, or just its inter-individual characteristic can potentially affect a single latency value without having significance for hearing.

### Triangle of Relationships and Cautionary Notes

According to the minimum-phase assumption, frequency tuning and latency are closely interrelated. In the triangle shown in Fig. 1, we purposely added the threshold. Starting from the passive cochlea, the hearing threshold might be expected in humans to correspond to approximately 60 dB SPL over a large range of frequencies, and comes along with broadly tuned traveling waves [58].

In the classic view, positive-feedback amplification of the outer hair cells is needed to reduce the threshold, thereby leading to narrow filter bandwidths. Originally, the mechanism responsible for that has been seen in suitably detuned tectorial-membrane resonance properties [59, 60]. While this view appeared to hold only in the basal part of the cochlea [61, 62], and is generally challenged with respect to the details of cochlear micromechanics [63], the general relations suggested by Fig. 1 are nonetheless supported by experimental and clinical evidence to a large extent. However, obviously they do not apply with ultimate strictness. For instance, the results shown in Fig. 8A,B demonstrate that a low threshold can be achieved even within one subject on the one ear with a notable level dependence, whereas on the contralateral side, there is almost no level dependence.

As we did not incorporate psychophysical and/or ABR measurements in this study, one can only speculate whether the frequency bandwidth of hearing follows these different level dependences. Either the minimum-phase property is violated, meaning that at low levels some ears add unnecessary delay to the one dictated by the minimum-phase principle (i.e., introduces an all pass), or the cochlea is able to achieve high amplification with clearly different filter shapes.

Given the scatter and confounds considered above when comparing pulsed DPOAE latencies to ABR data, determining the exact factor relating OAE backpropagation to forward propagation remains challenging. To accurately determine the appropriate factor for estimating forward delays from pulsed DPOAE latencies, further investigations are required. These could include experiments that simultaneously record ECochG or ABR data that tightly restrict the potential generating region where latency is measured.

## Conclusion

The general frequency dependence of the nonlinear-distortion component of the pulsed DPOAE latency derived by analyzing pulse responses in the time domain matched generally well with the results known from the literature examining the SFOAE [41, 42], governed by the coherent-reflection mechanism, and the coherent-reflection component of the DPOAE [41], where the upper frequency limit, however, was 5–8 kHz. This general agreement might be summarized as an approximate 0.3 dB/dB rise in periods with frequency. Breaks in the scaling law in the investigated frequency range from $f_2$ = 1–14 kHz are mostly not in agreement with those found by others, when relying on the phase gradient method, and when the underlying mechanism was coherent reflection. Better agreement with respect to changes in the frequency dependence is found in comparison to the coherent-reflection component of the DPOAE, as extracted by wavelet-transform-based time-frequency analysis [41].

Our data show that scaling symmetry may be clearly violated in certain frequency regions even in the presence of perfectly normal-hearing thresholds, if one is to set the limit for local scaling symmetry to a frequency exponent of $\le 0.3$, corresponding to a 23% change per octave. In contrast, the mean value of the latency in periods changes within the approximately half octave between 4 and 6 kHz by 28%.

The inconsistency between ABR latencies and OAE latencies at frequencies $\ge 4$ kHz remains unresolved; ABR latencies appear to generally predict higher latencies than OAE. Moreover, on the individual level, in some exceptional cases, high latencies appear not to be necessary to reach excellent hearing threshold, a property which might violate the minimum-phase predictions and warrant model studies to mimic such effects. The main findings thus might be summarized as follows:

- For frequencies above 1 kHz and up to 14 kHz, our data, as well as much of the literature on OAE latency, is commensurate with a scaling law of roughly 0.3 dB/dB.
- In our data appears to be a transition region between 4 and 6 kHz where the scaling law in an individual ear might approach 1 dB/dB (Fig. 4C) and thus appears to violate the assumption of local scaling symmetry.
- In individual cases (i.e., ear-frequency combinations), even in the presence of perfectly normal pure-tone threshold, level dependence can be very shallow, thus challenging the general notion that high gain can only be reached with long latencies.
- Pulsed DPOAE latency directly assessed in the time domain shows a high test-retest stability as reflected by relative absolute differences.
- The relation between ABR latency and OAE latency remains unresolved and appears to require a model of single-spike probability summation as well as transient cochlear waves, when stimulus ramps are used, which are considerably shorter than those used here.

## Supplementary Information

Below is the link to the electronic supplementary material.

Supplementary file 1 (docx 34 KB)

## Figures and Tables

**Fig. 11:**
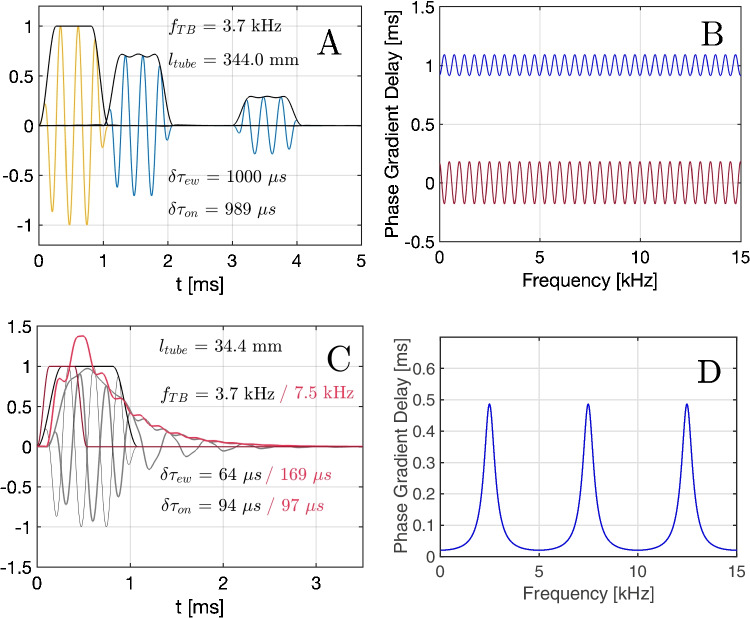
Simulation of latency determination in an acoustic tube. Each tone burst has a total duration of 4 periods, with one period allocated to both the onset and offset ramps. Left column: Time domain responses. Right column: Phase gradient delay. Upper row: Long tube. Bottom row: Short tube. For the short tube, only onset delay values ($\delta \tau _{on}$) provide a robust delay measure. The phase gradient method works well in the long tube, provided reflection in the tube is low

## Data Availability

Raw data and derived data can be made available upon personal request to the corresponding author.

## Appendix A: PGM Complexities

In physics and technology, latency is defined as the time delay required for a wave packet to travel a distance. The wave packet is also understood as a piece of information, in contrast to a continuous signal, such as sunlight, which permits theoretically only the determination of the phase velocity of light, but not its velocity. The measurement of the time delay of a wave packet, which in the case of the cochlea is a pulse or tone-burst response as presented here and elsewhere [26, 36, 71, 73, 74], might thus be regarded as the most direct means of measuring latency. In the case of nonlinear systems, it is generally maintained to be the only rigorous method (cf. [72], p. 172). According to linear systems theory, latency can also be inferred from the phase gradient of the system function $H(\omega )$. From here, the focus shifts from tone bursts to the measurement of *H* using quasi-continuous signals of discrete or continuously swept frequencies. Although this approach is common practice, complexities of this technique are rarely discussed in detail, even in systems where linearization is justifiable. Therefore, three exemplary cases are discussed in the following.

### Measurement of Latency in Case of Waves of Opposite Travel Direction

Consider a lossless tube with a length of $l_\textrm{tube}$=344 mm, and an inner diameter of 8 mm. The characteristic acoustic impedance is 820 $\Omega$ (sound velocity *c*=344 m/s, air density $\rho =1.2$ kg/m$^3$), terminated with $Z_\textrm{t}$. Figure 11A shows the stimulating tone pulse along with two pulse responses recorded at the far end of the tube ($Z_\textrm{t}=2000 \; \Omega$). As expected, the first pulse response is delayed by 1 ms. In the Figure, two delays are computed: (1) The delay from the half-maximum of the onset ramp of the stimulus pulse to the inflection point of the pulse response, referred to here as onset latency method, $\tau _\textrm{on}$. This is comparable to the OD method used in our work. (2) The energy-weighted delay, $\tau _{ew}$. Figure 11B shows the correspondent phase gradient for $Z_\textrm{t}=900 \Omega$. In the example shown in Fig. 11A, B, all methods perform well. However, when the reflectance of the termination is increased, interference effects between forward- and repeatedly back and forth travelled waves introduce errors in the phase-gradient method (PGM), as shown with $Z_\textrm{t}=2000 \Omega$ in the blue curve of Fig. 11B.

Figure 11C and D show the simulation for a short tube of 34.4 mm length, again with 2000 $\Omega$ termination, leading to considerable overlap of the multiple reflected waves. Figure 11C demonstrates for two different tone-burst frequencies, the interference states between the overlapping wave packets result in clearly different shapes of the pulse responses. The energy-weighted delay becomes less reliable once multiple reflections overlap with the first pulse response (Fig. 11C). In comparison, the onset latency method provides a more consistent delay estimate, staying within 6% of the expected 100 $\mu$. In contrast, the naive use of the PGM, depending on frequency, would — providing values between about 20 and 500 $\mu$s — lead to highly misleading results (Fig. 11D).

Note that as the latency measures were taken at the far end of the tube, the phase gradient in these examples never drops below zero, because, in a passive system, the backward-traveling wave can only be reflected with $\le$100%.

### Effect of Reflections on PGM Measurements at The Near Site

In the application of the PGM to the cochlea, the recording has to be done at the entrance of the system, and backward-traveling signals are analyzed. Figure 11B shows, for the long tube, the phase gradient delay derived from the input impedance of the tube (red curve). It is obvious that, in this configuration, the first pulse response would return after 2 ms, positioned between the two pulse responses at the far end of the tube (Fig. 11A, not shown). The key observation is that the PGM yields negative delays at frequencies where the backward traveling wave interferes destructively, thus delivering formally a non-causal result. One might object that in any OAE measurement, the forward-traveling stimulus, which in this example leads to spoofing the PGM, is effectively discarded before applying the PGM. However, it is important to recognize that any additional source within an OAE generation region can, in principle, produce similar interference phenomena that introduce errors into the PGM-based latency estimates. In this context, consider a scenario where outer-hair cell activity is (pathologically) reduced within part of the distributed emission-generating region, then two centers of activity would dominate the OAE. In case of a coherent-reflection process, the phase difference between these both centers is not predictable, and for the PGM, this would imply errors analogous to those illustrated by the examples above.

While latency determination in the time domain may also lead to complexities such as a deformed pulse response due to interference of two wave packets of different latency, this phenomenon can be identified as an unusually broadened or structured pulse response. On the other hand, the onset latency would remain approximately correct and would never formally indicate a non-causal event by taking on negative values.

### PGM and DPOAE

The application of the PGM to DPOAE is a peculiar case because the signal source is intracochlear. When sweeping a two-tone stimulus while keeping the frequency ratio constant, the coupled motion of both traveling waves with $f_1,\,f_2$ across the cochlea leads to an almost constant phase difference between both primaries at the $f_2$-place [75]. The latency, when naively derived from the phase gradient of the $f_\textrm{DP}$ signal, is then almost zero — the more the more the assumption of local scaling symmetry holds, and thus does not correspond to the true signal latency of this signal component. Actually, in this case, it has to be regarded as a proxy for cochlear dispersion [75]. The same applies to the phase-gradient latency of the long-latency component: This derived latency informs about the delay of the coherent-reflection component *relative* to the signal generated at the nonlinear-distortion place, not about its true latency with respect to the original stimulus in the ear canal, which is almost double of that. This shows that only *after* having understood the system, the PGM can be successfully used to derive the latency of one of the both components, i.e., of the coherent-reflection source.

### Conclusive Remarks

This appendix has not been written to disapprove cochlear latency results obtained by the PGM. Research on OAE delay using the PGM, including work cited herein, has undertaken numerous steps to validate its results. For researchers actively engaged in latency analysis using PGM, including comparison with cochlear models, the points raised by the above-given examples may be familiar. However, this discussion may be helpful for those who apply the PGM more generally, without delving into its underlying assumptions. In any case, it is worth recognizing that the issue of apparent non-causality, as discussed in [70], does not arise by definition in direct delay measurements — as long as one is not using unappropriate filtering techniques.

## Appendix B: Parameters of The Cochlea Model

Simulations of DPOAE responses in the present work were conducted using a cochlear model based on mechanically and longitudinally coupled oscillators driven by hydrodynamic coupling terms from stapes and basilar-membrane motion [64, 76, 77]. The model was adapted to represent the anatomical and biomechanical characteristics of the human cochlea and extended by a simplified middle-ear model, implemented as a three-mass oscillator approximating the human middle-ear transfer function (Table 4). This extension allows the solution of the equations of motion directly in the time domain. For a summary of the governing equations, the reader is referred to [52], and, for a detailed derivation and discussion, to [20]. The following section lists the model parameters that were used in the present study to implement the equations of motion [52].

**Table 4:** Values for the mechanical parameters $m_i$ (mg), $d_i$ (kg s$^{-1}$), and $k_i$ (kg s$^{-2}$) of the three-mass oscillator representing the middle-ear transfer function

Mass			Damping			Stiffness	
$m_1$	18.1		$d_1$	0.017		$k_1$	75.1
$m_2$	17.0		$d_2$	0		$k_2$	2785.4
$m_3$	2.9		$d_3$	0		$k_3$	9424.5
			$d_4$	0.056		$k_4$	3896.0
			$d_{\text {C}}$	0.35			

$d_\text {C}$ denotes an additional damping term substituting the cochlear load and should be set to zero once the cochlear and middle-ear models are coupled

The place-dependent mechanical properties of the basilar membrane (BM) are specified by the following equations:

B1 $$\begin{aligned} m(x) = m_0b_{\text {BM}}(x) \end{aligned}$$

B2 $$\begin{aligned} d(x) = \alpha _{\text {d}} \eta _{\text {E}} \frac{b_{\text {BM}} (x)}{l_{\text {stereo}(x)}} \end{aligned}$$

B3 $$\begin{aligned} s(x) = \eta _{\text {C}}b_{\text {BM}}(x)h_{\text {CO}}(x) \end{aligned}$$

B4 $$\begin{aligned} k(x) = k_0 \cdot 10^{-\gamma _kx} \end{aligned}$$

where $b_{\text {BM}}(x)$ and $h_{\text {CO}}(x)$ are the width of the BM and the height of the organ of Corti, respectively. *x* is the longitudinal coordinate normalized to the length of the cochlea, which was chosen to be 3.5 cm in the present work. Table 5 lists the model parameters used to set the BM properties. Here, the stiffness of the BM was fitted to match the tonotopic frequency map of the human cochlea for frequencies from 0.125 to 10 kHz [21].

**Table 5:** Model parameters to specify the mechanical properties of the basilar membrane

Model parameter	Value	Unit
$m_0$	$2.47 \cdot 10^{-2}$	kg m$^{-2}$
$\eta _{\text {E}}$	$1.5 \cdot 10^{-3}$	kg m$^{-1}$ s$^{-1}$
$\eta _{\text {C}}$	$1.5 \cdot 10^{-2}$	kg m$^{-1}$ s$^{-1}$
$\alpha _{\text {d}}$	1.05	-
$l_{\text {stereo}}$	2 – 6	$\mu$m
$k_0$	$1.2 \cdot 10^5$	kg m$^{-1}$ s$^{-2}$
$\gamma _k$	7.96	-

Note, the length of the stereocilia $l_{\text {stereo}}$ increases from base to apex

**Table 6:** Values of the model parameters defining the mechanical properties of the tectorial membrane

Model parameter	Value	Unit
$\eta _{\text {TM}}$	0.18	kg m$^{-1}$ s$^{-1}$
$r_{\text {CO/TM}}$	2.5	-

The model parameters of the mechanical properties of the tectorial membrane (TM) are a function of *x* and specified by the following equations:

B5 $$\begin{aligned} \omega _{\text {TM}}(x) = 2\pi f_{\text {CF}}(x) \cdot 2^{-0.5} \end{aligned}$$

B6 $$\begin{aligned} \gamma _{\text {TM}}(x) = 2D_{\text {TM}}(x)\omega _{\text {TM}}(x) \end{aligned}$$

B7 $$\begin{aligned} D_{\text {TM}}(x) = \frac{d(x)}{2m_{\text {TM}}(x) \omega _{\text {TM}}(x)} \end{aligned}$$

B8 $$\begin{aligned} m_{\text {TM}}(x) = \frac{1}{r_{\text {CO/TM}}} m(x) \end{aligned}$$

B9 $$\begin{aligned} s_{\text {TM}}(x) = A_{\text {TM}}(x) \eta _{\text {TM}} \end{aligned}$$

where $f_{\text {CF}}(x)$ represents the characteristic frequency along the BM. The TM cross section area is derived from the BM anatomy by

B10 $$\begin{aligned} A_{\text {TM}}(x) = \frac{1}{r_{\text {CO/TM}}} b_{\text {BM}}(x)h_{\text {CO}}(x) \end{aligned}$$

with the apical cross-section ratio $r_{\text {CO/TM}}$ (see Table 6).

The cochlear force density $F_{\text {CA}}$ compensates the viscous damping and is scaled via the empirical gain function

B11 $$\begin{aligned} \lambda (x) = \kappa _1 \left( 1 - e^{- \kappa _2 x} \right) + \frac{\kappa _3}{\sqrt{2 \pi \kappa _4}} e^{ - \frac{\left( x- \kappa _5 \right) ^2}{2 {\kappa _4} ^2}} \end{aligned}$$

which adjusts the amplification provided by the model to values derived from frequency tuning curves of the human cochlea [15]. The saturation of the sigmoidal nonlinearity of the cochlear amplifier (cf. [77]) was scaled according to the longitudinal variation in stereocilia length:

B12 $$\begin{aligned} a(x) = 1/(a_0e^{\log (1/3)x}) \end{aligned}$$

Table 7 lists the model parameters and coefficients of Eq. B11 to determine the cochlear force density.

**Table 7:** Model parameters and coefficients to define the nonlinear cochlear amplification

Model parameter	Value	Unit
$\beta$	0.927	-
$a_0$	15	nm
$\kappa _1$	1.27	-
$\kappa _2$	30.0	-
$\kappa _3$	0.38	-
$\kappa _4$	0.21	-
$\kappa _5$	0.175	-

## Footnotes

1 This model [20] does not include a threshold in the strict sense. However, the gain — set to compensate the damping caused by the cochlear fluids — relates OHC force to stereocilia displacement and yields a compression region in basilar membrane vibration growth functions. Depending on frequency, the onset of the compression region occurs around 30–40 dB SPL, with a transition to passive behavior between 70 and 80 dB SPL. A value of about 20 dB below the start of the compression region might be seen as a proxy for the threshold within the framework of a cochlea model without neural interface.

2 The term scaling symmetry means that traveling waves of different frequency have identical shape when shifted along a logarithmic scale. Under this premise, tuning is independent of frequency, and the exponent of frequency dependence of latency in terms of periods is zero. For instance, in chinchilla, scaling symmetry is approximately valid above 6 kHz [31], whereas in humans, at least up to 8 kHz, it is not [17].

3 Rasetshwane et al. [27] give the total width of their pulse; from there, we computed the Blackman window with a=0.16 and 4 ms window size.

## Code Availability

The cochlea model will be made available alongside an upcoming paper that describes it in detail.
