# Supplementary material for: Latencies of Pulsed Distortion-Product Otoacoustic Emissions and Their Relation to Auditory Brainstem Responses
Source: J Assoc Res Otolaryngol. 2025 Nov 26;27(1):83–105. doi: 10.1007/s10162-025-01019-7 (PMC12948746; doi:10.1007/s10162-025-01019-7)
Supplement: Supplementary file 1 — (docx 34 KB) [file 10162_2025_1019_MOESM1_ESM.docx]

**Table 1.** Dependence on f_2_ and L_2_ ID. Test-retest reliability of pulsed DPOAE latencies of the nonlinear-distortion component extracted with onset decomposition (τ in ms), the corresponding number of periods in their dimensionless form (N) and the number of periods scaled in dB $\Gamma$ for each stimulus frequency f_2_ and stimulus level L_2_. N denotes the number of ADs. The test-retest reliability was ascertained with the median of absolute differences (AD), their interquartile range (IQR), and their 90% range of data. The 90% range of AD may serve as a clinical reference to detect pathologic test-retest differences.

|  |  |  | $\boldsymbol{\Gamma}$ **(dB)** | | |  | **τ (ms)** | | |  | ***N*** | | |  |  |
| --- | --- | --- | --- | --- | --- | --- | --- | --- | --- | --- | --- | --- | --- | --- | --- |
| ***f*_2_ (kHz)** | ***L*_2_ (dB SPL)** |  | **AD median** | **IQR** | **90% range** |  | **AD median** | **IQR** | **90% range** |  | **AD median** | **IQR** | **90% range** |  | **N** |
|  |  |  |  |  |  |  |  |  |  |  |  |  |  |  |  |
| 1 | 25 |  | 1.49 | 2.28 | 6.46 |  | 2.28 | 3.41 | 12.85 |  | 2.28 | 3.41 | 12.85 |  | 15 |
| 1 | 30 |  | 1.30 | 1.51 | 2.78 |  | 1.71 | 1.78 | 3.98 |  | 1.71 | 1.78 | 3.98 |  | 42 |
| 1 | 35 |  | 0.97 | 1.28 | 2.42 |  | 1.40 | 1.62 | 3.87 |  | 1.40 | 1.62 | 3.87 |  | 95 |
| 1 | 40 |  | 0.80 | 1.14 | 2.98 |  | 1.15 | 1.58 | 3.36 |  | 1.15 | 1.58 | 3.36 |  | 159 |
| 1 | 45 |  | 0.99 | 1.37 | 2.93 |  | 1.34 | 1.82 | 4.23 |  | 1.34 | 1.82 | 4.23 |  | 228 |
| 1 | 50 |  | 0.81 | 1.18 | 3.71 |  | 1.06 | 1.38 | 4.31 |  | 1.06 | 1.38 | 4.31 |  | 248 |
| 1 | 55 |  | 0.61 | 1.21 | 3.09 |  | 0.78 | 1.37 | 3.63 |  | 0.78 | 1.37 | 3.63 |  | 248 |
| 1 | 60 |  | 0.63 | 1.24 | 2.94 |  | 0.81 | 1.59 | 3.94 |  | 0.81 | 1.59 | 3.94 |  | 270 |
| 1 | 65 |  | 0.63 | 1.12 | 3.01 |  | 0.76 | 1.07 | 3.32 |  | 0.76 | 1.07 | 3.32 |  | 260 |
| 1 | 70 |  | 0.70 | 1.45 | 5.28 |  | 0.90 | 1.52 | 4.14 |  | 0.90 | 1.52 | 4.14 |  | 255 |
|  |  |  |  |  |  |  |  |  |  |  |  |  |  |  |  |
| 1.5 | 25 |  | 1.00 | 1.59 | 5.09 |  | 1.28 | 1.60 | 6.92 |  | 1.92 | 2.39 | 10.38 |  | 60 |
| 1.5 | 30 |  | 0.72 | 1.04 | 2.47 |  | 0.89 | 1.19 | 2.79 |  | 1.33 | 1.79 | 4.18 |  | 126 |
| 1.5 | 35 |  | 0.69 | 0.98 | 1.99 |  | 0.78 | 1.18 | 2.10 |  | 1.17 | 1.77 | 3.15 |  | 167 |
| 1.5 | 40 |  | 0.67 | 1.09 | 2.40 |  | 0.68 | 1.13 | 2.32 |  | 1.02 | 1.69 | 3.49 |  | 242 |
| 1.5 | 45 |  | 0.54 | 0.88 | 1.81 |  | 0.54 | 0.88 | 1.89 |  | 0.80 | 1.32 | 2.83 |  | 276 |
| 1.5 | 50 |  | 0.61 | 0.81 | 2.08 |  | 0.59 | 0.77 | 1.79 |  | 0.89 | 1.15 | 2.68 |  | 363 |
| 1.5 | 55 |  | 0.65 | 0.96 | 1.84 |  | 0.59 | 0.80 | 1.67 |  | 0.89 | 1.20 | 2.50 |  | 347 |
| 1.5 | 60 |  | 0.68 | 0.96 | 2.04 |  | 0.62 | 0.87 | 1.71 |  | 0.92 | 1.30 | 2.57 |  | 368 |
| 1.5 | 65 |  | 0.57 | 0.86 | 1.81 |  | 0.51 | 0.81 | 1.52 |  | 0.77 | 1.22 | 2.28 |  | 359 |
| 1.5 | 70 |  | 0.52 | 0.76 | 1.87 |  | 0.50 | 0.72 | 1.66 |  | 0.75 | 1.08 | 2.49 |  | 399 |
|  |  |  |  |  |  |  |  |  |  |  |  |  |  |  |  |
| 2 | 25 |  | 1.08 | 1.54 | 2.99 |  | 0.85 | 1.10 | 3.55 |  | 1.69 | 2.21 | 7.10 |  | 34 |
| 2 | 30 |  | 0.90 | 1.15 | 2.25 |  | 0.90 | 1.24 | 2.43 |  | 1.80 | 2.48 | 4.86 |  | 99 |
| 2 | 35 |  | 0.53 | 0.77 | 3.02 |  | 0.50 | 0.62 | 2.04 |  | 1.00 | 1.24 | 4.08 |  | 145 |
| 2 | 40 |  | 0.49 | 0.83 | 1.72 |  | 0.41 | 0.65 | 1.37 |  | 0.82 | 1.29 | 2.74 |  | 214 |
| 2 | 45 |  | 0.42 | 0.72 | 1.64 |  | 0.33 | 0.59 | 1.22 |  | 0.66 | 1.18 | 2.44 |  | 250 |
| 2 | 50 |  | 0.53 | 0.84 | 1.92 |  | 0.40 | 0.55 | 1.40 |  | 0.79 | 1.10 | 2.81 |  | 296 |
| 2 | 55 |  | 0.46 | 0.79 | 1.80 |  | 0.33 | 0.56 | 1.28 |  | 0.66 | 1.12 | 2.56 |  | 333 |
| 2 | 60 |  | 0.53 | 0.81 | 1.87 |  | 0.37 | 0.55 | 1.33 |  | 0.74 | 1.11 | 2.65 |  | 338 |
| 2 | 65 |  | 0.52 | 0.97 | 2.00 |  | 0.37 | 0.66 | 1.29 |  | 0.74 | 1.33 | 2.57 |  | 334 |
| 2 | 70 |  | 0.54 | 0.76 | 2.04 |  | 0.40 | 0.57 | 1.39 |  | 0.80 | 1.14 | 2.78 |  | 358 |
|  |  |  |  |  |  |  |  |  |  |  |  |  |  |  |  |
| 3 | 25 |  | 1.29 | 1.93 | 3.86 |  | 0.74 | 1.02 | 2.21 |  | 2.22 | 3.06 | 6.64 |  | 38 |
| 3 | 30 |  | 0.69 | 0.98 | 1.99 |  | 0.45 | 0.57 | 1.23 |  | 1.35 | 1.71 | 3.68 |  | 110 |
| 3 | 35 |  | 0.66 | 0.69 | 1.80 |  | 0.40 | 0.47 | 1.16 |  | 1.20 | 1.41 | 3.48 |  | 154 |
| 3 | 40 |  | 0.54 | 0.77 | 1.42 |  | 0.31 | 0.46 | 0.83 |  | 0.92 | 1.38 | 2.49 |  | 232 |
| 3 | 45 |  | 0.79 | 0.90 | 1.86 |  | 0.42 | 0.48 | 1.06 |  | 1.26 | 1.44 | 3.17 |  | 280 |
| 3 | 50 |  | 0.79 | 0.99 | 2.19 |  | 0.40 | 0.47 | 1.17 |  | 1.20 | 1.41 | 3.51 |  | 306 |
| 3 | 55 |  | 1.00 | 1.14 | 2.64 |  | 0.48 | 0.59 | 1.43 |  | 1.44 | 1.77 | 4.28 |  | 345 |
| 3 | 60 |  | 0.71 | 1.15 | 2.74 |  | 0.34 | 0.60 | 1.50 |  | 1.02 | 1.80 | 4.51 |  | 356 |
| 3 | 65 |  | 0.62 | 1.02 | 2.24 |  | 0.31 | 0.52 | 1.30 |  | 0.92 | 1.56 | 3.91 |  | 346 |
| 3 | 70 |  | 0.62 | 0.80 | 1.79 |  | 0.32 | 0.42 | 1.06 |  | 0.96 | 1.26 | 3.17 |  | 357 |
|  |  |  |  |  |  |  |  |  |  |  |  |  |  |  |  |
| 4 | 30 |  | 0.65 | 0.76 | 1.98 |  | 0.38 | 0.47 | 1.29 |  | 1.50 | 1.88 | 5.16 |  | 78 |
| 4 | 35 |  | 0.40 | 0.68 | 1.47 |  | 0.23 | 0.34 | 0.74 |  | 0.92 | 1.36 | 2.97 |  | 116 |
| 4 | 40 |  | 0.61 | 0.87 | 1.80 |  | 0.30 | 0.45 | 0.99 |  | 1.18 | 1.80 | 3.97 |  | 146 |
| 4 | 45 |  | 0.51 | 0.67 | 1.22 |  | 0.25 | 0.31 | 0.58 |  | 1.00 | 1.24 | 2.30 |  | 196 |
| 4 | 50 |  | 0.53 | 0.71 | 1.68 |  | 0.24 | 0.30 | 0.77 |  | 0.94 | 1.20 | 3.08 |  | 258 |
| 4 | 55 |  | 0.58 | 0.78 | 1.73 |  | 0.27 | 0.34 | 0.76 |  | 1.08 | 1.36 | 3.04 |  | 313 |
| 4 | 60 |  | 0.66 | 0.95 | 1.84 |  | 0.29 | 0.39 | 0.82 |  | 1.14 | 1.56 | 3.28 |  | 348 |
| 4 | 65 |  | 0.66 | 0.97 | 2.06 |  | 0.28 | 0.42 | 0.91 |  | 1.12 | 1.68 | 3.62 |  | 396 |
| 4 | 70 |  | 0.68 | 1.08 | 2.33 |  | 0.26 | 0.41 | 0.93 |  | 1.02 | 1.64 | 3.71 |  | 402 |
| 4 | 75 |  | 0.65 | 1.00 | 2.06 |  | 0.24 | 0.38 | 0.85 |  | 0.96 | 1.52 | 3.40 |  | 414 |
|  |  |  |  |  |  |  |  |  |  |  |  |  |  |  |  |
| 5 | 30 |  | 1.25 | 2.32 | 8.14 |  | 0.67 | 1.26 | 3.05 |  | 3.35 | 6.30 | 15.25 |  | 39 |
| 5 | 35 |  | 0.78 | 1.82 | 7.88 |  | 0.39 | 0.91 | 2.31 |  | 1.95 | 4.55 | 11.54 |  | 67 |
| 5 | 40 |  | 0.86 | 1.14 | 2.26 |  | 0.43 | 0.56 | 1.00 |  | 2.15 | 2.80 | 5.00 |  | 148 |
| 5 | 45 |  | 0.79 | 1.09 | 2.21 |  | 0.34 | 0.54 | 1.05 |  | 1.68 | 2.70 | 5.25 |  | 204 |
| 5 | 50 |  | 0.79 | 1.14 | 2.70 |  | 0.36 | 0.52 | 1.20 |  | 1.80 | 2.60 | 5.98 |  | 267 |
| 5 | 55 |  | 0.93 | 1.36 | 2.95 |  | 0.39 | 0.55 | 1.12 |  | 1.95 | 2.75 | 5.60 |  | 302 |
| 5 | 60 |  | 0.90 | 1.38 | 2.59 |  | 0.33 | 0.53 | 1.07 |  | 1.65 | 2.65 | 5.36 |  | 347 |
| 5 | 65 |  | 0.86 | 1.15 | 2.39 |  | 0.34 | 0.44 | 0.89 |  | 1.68 | 2.20 | 4.47 |  | 386 |
| 5 | 70 |  | 0.84 | 1.41 | 2.66 |  | 0.30 | 0.52 | 1.05 |  | 1.50 | 2.60 | 5.25 |  | 399 |
| 5 | 75 |  | 0.71 | 1.37 | 3.28 |  | 0.25 | 0.51 | 1.23 |  | 1.25 | 2.55 | 6.15 |  | 403 |
|  |  |  |  |  |  |  |  |  |  |  |  |  |  |  |  |
| 6 | 30 |  | 1.18 | 1.34 | 6.30 |  | 0.54 | 0.74 | 3.90 |  | 3.24 | 4.44 | 23.39 |  | 65 |
| 6 | 35 |  | 0.97 | 1.67 | 6.39 |  | 0.46 | 0.83 | 4.04 |  | 2.76 | 4.98 | 24.26 |  | 100 |
| 6 | 40 |  | 1.05 | 1.59 | 3.24 |  | 0.46 | 0.68 | 1.28 |  | 2.76 | 4.08 | 7.66 |  | 123 |
| 6 | 45 |  | 1.01 | 1.60 | 3.01 |  | 0.45 | 0.60 | 1.16 |  | 2.67 | 3.60 | 6.94 |  | 186 |
| 6 | 50 |  | 1.04 | 1.69 | 3.93 |  | 0.42 | 0.66 | 1.40 |  | 2.49 | 3.96 | 8.39 |  | 270 |
| 6 | 55 |  | 0.66 | 1.11 | 2.41 |  | 0.25 | 0.42 | 0.94 |  | 1.50 | 2.52 | 5.64 |  | 289 |
| 6 | 60 |  | 0.77 | 1.22 | 2.74 |  | 0.28 | 0.43 | 1.01 |  | 1.68 | 2.58 | 6.06 |  | 319 |
| 6 | 65 |  | 0.79 | 1.37 | 4.01 |  | 0.28 | 0.48 | 1.18 |  | 1.68 | 2.88 | 7.08 |  | 337 |
| 6 | 70 |  | 0.66 | 1.14 | 2.57 |  | 0.23 | 0.38 | 0.89 |  | 1.38 | 2.28 | 5.34 |  | 324 |
| 6 | 75 |  | 0.79 | 1.16 | 2.72 |  | 0.27 | 0.38 | 1.01 |  | 1.62 | 2.28 | 6.07 |  | 373 |
|  |  |  |  |  |  |  |  |  |  |  |  |  |  |  |  |
| 8 | 30 |  | 0.60 | 0.58 | 2.56 |  | 0.22 | 0.21 | 0.77 |  | 1.76 | 1.68 | 6.14 |  | 50 |
| 8 | 35 |  | 0.86 | 1.06 | 2.11 |  | 0.29 | 0.39 | 0.81 |  | 2.32 | 3.12 | 6.48 |  | 94 |
| 8 | 40 |  | 0.70 | 0.98 | 2.80 |  | 0.23 | 0.32 | 0.90 |  | 1.80 | 2.56 | 7.19 |  | 122 |
| 8 | 45 |  | 0.63 | 0.93 | 1.88 |  | 0.20 | 0.28 | 0.60 |  | 1.60 | 2.24 | 4.76 |  | 144 |
| 8 | 50 |  | 0.86 | 1.14 | 2.81 |  | 0.26 | 0.32 | 0.88 |  | 2.08 | 2.56 | 7.06 |  | 206 |
| 8 | 55 |  | 0.65 | 0.85 | 1.92 |  | 0.19 | 0.25 | 0.58 |  | 1.52 | 2.00 | 4.64 |  | 239 |
| 8 | 60 |  | 0.69 | 1.08 | 2.62 |  | 0.19 | 0.32 | 0.80 |  | 1.52 | 2.56 | 6.40 |  | 239 |
| 8 | 65 |  | 0.64 | 1.09 | 3.21 |  | 0.19 | 0.30 | 1.00 |  | 1.52 | 2.40 | 7.98 |  | 262 |
| 8 | 70 |  | 0.72 | 1.31 | 4.95 |  | 0.20 | 0.34 | 1.12 |  | 1.60 | 2.72 | 8.96 |  | 273 |
| 8 | 75 |  | 0.85 | 1.44 | 3.61 |  | 0.23 | 0.41 | 0.91 |  | 1.84 | 3.28 | 7.30 |  | 275 |
|  |  |  |  |  |  |  |  |  |  |  |  |  |  |  |  |
| 9 | 30 |  | 1.24 | 1.81 | 3.79 |  | 0.41 | 0.53 | 1.15 |  | 3.69 | 4.77 | 10.31 |  | 62 |
| 9 | 35 |  | 0.81 | 1.22 | 2.85 |  | 0.26 | 0.42 | 0.81 |  | 2.34 | 3.78 | 7.29 |  | 139 |
| 9 | 40 |  | 0.77 | 1.03 | 2.56 |  | 0.24 | 0.29 | 0.67 |  | 2.16 | 2.61 | 6.07 |  | 155 |
| 9 | 45 |  | 0.79 | 0.79 | 1.94 |  | 0.21 | 0.25 | 0.55 |  | 1.89 | 2.25 | 4.93 |  | 181 |
| 9 | 50 |  | 0.83 | 1.21 | 2.54 |  | 0.24 | 0.35 | 0.75 |  | 2.16 | 3.15 | 6.75 |  | 218 |
| 9 | 55 |  | 0.75 | 1.16 | 2.57 |  | 0.21 | 0.35 | 0.67 |  | 1.89 | 3.15 | 5.99 |  | 214 |
| 9 | 60 |  | 0.76 | 1.21 | 2.87 |  | 0.20 | 0.34 | 0.81 |  | 1.80 | 3.06 | 7.29 |  | 239 |
| 9 | 65 |  | 0.92 | 1.32 | 2.45 |  | 0.22 | 0.34 | 0.72 |  | 1.98 | 3.06 | 6.44 |  | 241 |
| 9 | 70 |  | 0.83 | 1.42 | 3.01 |  | 0.21 | 0.41 | 0.93 |  | 1.89 | 3.69 | 8.37 |  | 259 |
| 9 | 75 |  | 0.89 | 1.60 | 4.17 |  | 0.23 | 0.44 | 0.86 |  | 2.07 | 3.96 | 7.70 |  | 273 |
|  |  |  |  |  |  |  |  |  |  |  |  |  |  |  |  |
| 10 | 35 |  | 1.29 | 1.40 | 3.81 |  | 0.43 | 0.41 | 1.05 |  | 4.30 | 4.10 | 10.50 |  | 49 |
| 10 | 40 |  | 1.37 | 1.61 | 3.24 |  | 0.38 | 0.42 | 0.89 |  | 3.80 | 4.20 | 8.88 |  | 115 |
| 10 | 45 |  | 0.80 | 1.14 | 3.13 |  | 0.21 | 0.31 | 0.71 |  | 2.05 | 3.10 | 7.13 |  | 188 |
| 10 | 50 |  | 0.83 | 1.19 | 2.45 |  | 0.21 | 0.29 | 0.62 |  | 2.10 | 2.90 | 6.19 |  | 220 |
| 10 | 55 |  | 0.75 | 1.23 | 2.60 |  | 0.19 | 0.34 | 0.72 |  | 1.90 | 3.40 | 7.20 |  | 233 |
| 10 | 60 |  | 0.60 | 0.92 | 2.77 |  | 0.15 | 0.24 | 0.69 |  | 1.50 | 2.40 | 6.90 |  | 244 |
| 10 | 65 |  | 0.76 | 1.21 | 3.01 |  | 0.19 | 0.28 | 0.86 |  | 1.90 | 2.80 | 8.60 |  | 265 |
| 10 | 70 |  | 0.68 | 0.68 | 2.56 |  | 0.16 | 0.22 | 0.59 |  | 1.60 | 2.20 | 5.90 |  | 242 |
| 10 | 75 |  | 0.96 | 1.48 | 3.40 |  | 0.23 | 0.39 | 0.79 |  | 2.30 | 3.90 | 7.87 |  | 262 |
| 10 | 80 |  | 1.06 | 1.45 | 3.39 |  | 0.26 | 0.39 | 0.78 |  | 2.60 | 3.90 | 7.84 |  | 255 |
|  |  |  |  |  |  |  |  |  |  |  |  |  |  |  |  |
| 11 | 35 |  | 1.16 | 1.54 | 2.68 |  | 0.37 | 0.50 | 0.82 |  | 4.07 | 5.50 | 9.02 |  | 29 |
| 11 | 40 |  | 0.94 | 1.01 | 2.11 |  | 0.26 | 0.26 | 0.61 |  | 2.86 | 2.86 | 6.72 |  | 78 |
| 11 | 45 |  | 0.70 | 0.93 | 2.60 |  | 0.20 | 0.27 | 0.59 |  | 2.20 | 2.97 | 6.47 |  | 100 |
| 11 | 50 |  | 0.88 | 0.96 | 2.50 |  | 0.23 | 0.27 | 0.62 |  | 2.53 | 2.97 | 6.84 |  | 148 |
| 11 | 55 |  | 0.77 | 1.32 | 2.57 |  | 0.20 | 0.34 | 0.62 |  | 2.20 | 3.74 | 6.82 |  | 185 |
| 11 | 60 |  | 0.81 | 1.25 | 3.11 |  | 0.20 | 0.29 | 0.65 |  | 2.20 | 3.19 | 7.15 |  | 213 |
| 11 | 65 |  | 0.74 | 1.16 | 2.66 |  | 0.17 | 0.29 | 0.63 |  | 1.82 | 3.19 | 6.90 |  | 252 |
| 11 | 70 |  | 0.81 | 1.26 | 3.54 |  | 0.19 | 0.31 | 0.78 |  | 2.09 | 3.41 | 8.53 |  | 254 |
| 11 | 75 |  | 0.99 | 1.39 | 3.17 |  | 0.23 | 0.33 | 0.70 |  | 2.53 | 3.63 | 7.74 |  | 247 |
| 11 | 80 |  | 0.77 | 1.40 | 2.87 |  | 0.19 | 0.33 | 0.66 |  | 2.04 | 3.63 | 7.30 |  | 210 |
|  |  |  |  |  |  |  |  |  |  |  |  |  |  |  |  |
| 12 | 35 |  |  |  |  |  |  |  |  |  |  |  |  |  | 0 |
| 12 | 40 |  |  |  |  |  |  |  |  |  |  |  |  |  | 1 |
| 12 | 45 |  | 0.58 | 0.94 | 3.42 |  | 0.15 | 0.22 | 0.92 |  | 1.80 | 2.64 | 11.04 |  | 18 |
| 12 | 50 |  | 1.10 | 1.18 | 2.37 |  | 0.30 | 0.28 | 0.59 |  | 3.60 | 3.36 | 7.03 |  | 83 |
| 12 | 55 |  | 1.15 | 1.37 | 2.86 |  | 0.28 | 0.36 | 0.67 |  | 3.30 | 4.32 | 8.00 |  | 112 |
| 12 | 60 |  | 0.88 | 1.30 | 2.71 |  | 0.21 | 0.32 | 0.73 |  | 2.52 | 3.84 | 8.76 |  | 169 |
| 12 | 65 |  | 0.72 | 1.12 | 2.43 |  | 0.16 | 0.26 | 0.55 |  | 1.92 | 3.12 | 6.61 |  | 178 |
| 12 | 70 |  | 0.71 | 0.93 | 1.75 |  | 0.15 | 0.20 | 0.41 |  | 1.80 | 2.40 | 4.86 |  | 194 |
| 12 | 75 |  | 0.67 | 1.05 | 2.59 |  | 0.15 | 0.21 | 0.52 |  | 1.80 | 2.52 | 6.20 |  | 182 |
| 12 | 80 |  | 0.59 | 1.05 | 2.09 |  | 0.12 | 0.22 | 0.52 |  | 1.44 | 2.64 | 6.24 |  | 177 |
|  |  |  |  |  |  |  |  |  |  |  |  |  |  |  |  |
| 13 | 35 |  |  |  |  |  |  |  |  |  |  |  |  |  | 0 |
| 13 | 40 |  | 1.27 | 1.92 | 2.53 |  | 0.32 | 0.39 | 0.62 |  | 4.16 | 5.07 | 8.11 |  | 17 |
| 13 | 45 |  | 0.63 | 0.81 | 1.43 |  | 0.15 | 0.19 | 0.34 |  | 1.95 | 2.47 | 4.42 |  | 56 |
| 13 | 50 |  | 0.88 | 1.11 | 2.55 |  | 0.17 | 0.26 | 0.54 |  | 2.21 | 3.38 | 7.02 |  | 103 |
| 13 | 55 |  | 0.98 | 1.27 | 3.51 |  | 0.20 | 0.27 | 0.69 |  | 2.60 | 3.51 | 9.02 |  | 133 |
| 13 | 60 |  | 0.90 | 1.24 | 2.97 |  | 0.19 | 0.26 | 0.56 |  | 2.47 | 3.38 | 7.24 |  | 152 |
| 13 | 65 |  | 0.74 | 1.41 | 3.39 |  | 0.13 | 0.23 | 0.56 |  | 1.69 | 2.99 | 7.24 |  | 162 |
| 13 | 70 |  | 0.62 | 0.88 | 2.43 |  | 0.11 | 0.15 | 0.47 |  | 1.43 | 1.95 | 6.08 |  | 163 |
| 13 | 75 |  | 0.61 | 0.88 | 2.10 |  | 0.12 | 0.16 | 0.35 |  | 1.56 | 2.08 | 4.55 |  | 125 |
| 13 | 80 |  | 0.68 | 0.83 | 1.95 |  | 0.13 | 0.15 | 0.39 |  | 1.69 | 1.95 | 5.01 |  | 124 |
|  |  |  |  |  |  |  |  |  |  |  |  |  |  |  |  |
| 14 | 35 |  | 0.47 | 0.64 | NA |  | 0.13 | 0.17 | NA |  | 1.75 | 2.38 | NA |  | 6 |
| 14 | 40 |  | 1.69 | 2.63 | 3.38 |  | 0.43 | 0.69 | 0.85 |  | 5.95 | 9.66 | 11.90 |  | 10 |
| 14 | 45 |  | 0.49 | 0.52 | 0.96 |  | 0.12 | 0.15 | 0.22 |  | 1.61 | 2.10 | 3.08 |  | 30 |
| 14 | 50 |  | 1.21 | 2.09 | 8.06 |  | 0.24 | 0.41 | 1.48 |  | 3.36 | 5.74 | 20.78 |  | 53 |
| 14 | 55 |  | 1.19 | 2.01 | 3.93 |  | 0.25 | 0.36 | 0.68 |  | 3.43 | 5.04 | 9.52 |  | 60 |
| 14 | 60 |  | 1.61 | 2.50 | 4.46 |  | 0.29 | 0.47 | 0.96 |  | 4.06 | 6.58 | 13.44 |  | 78 |
| 14 | 65 |  | 1.43 | 2.18 | 4.97 |  | 0.29 | 0.42 | 0.90 |  | 3.99 | 5.88 | 12.60 |  | 94 |
| 14 | 70 |  | 1.94 | 1.97 | 5.21 |  | 0.33 | 0.38 | 0.90 |  | 4.62 | 5.32 | 12.53 |  | 88 |
| 14 | 75 |  | 2.05 | 2.50 | 5.94 |  | 0.35 | 0.50 | 1.11 |  | 4.90 | 7.00 | 15.48 |  | 93 |
| 14 | 80 |  | 1.64 | 1.96 | 4.23 |  | 0.26 | 0.35 | 0.79 |  | 3.64 | 4.90 | 11.06 |  | 79 |
